# Overview of Ultrasound Biomicroscopy

**DOI:** 10.5005/jp-journals-10008-1105

**Published:** 2012-10-16

**Authors:** Mingguang He, Dandan Wang, Yuzheng Jiang

**Affiliations:** 1 Zhongshan Ophthalmic Center, Sun Yat-sen University, Guangzhou, China; 2 Zhongshan Ophthalmic Center, Sun Yat-sen University, Guangzhou, China; 3 Zhongshan Ophthalmic Center, Sun Yat-sen University, Guangzhou, China

**Keywords:** Ultrasound biomicroscopy, Scleral spur.

## Abstract

Ultrasound biomicroscopy (UBM) is a high-resolution ultrasound technique that allows noninvasive *in vivo* imaging of structural details of the anterior ocular segment at near light microscopic resolution and provides detailed assessment of anterior segment structures, including those obscured by normal anatomic and pathologic relations. This review gives an overview regarding the instrument, technique and its applications.

## HISTORY OF DEVELOPMENT

Ultrasound biomicroscopy (UBM) was first developed by Pavlin’s group in Canada over 10 years ago (Pavlin, Sherar, et al, 1990). Because it can provide images of the tissues and structures *in vivo* at microscopic resolution, similar to optical biomicroscopy, Pavlin’s group termed it ‘ultrasound biomicroscopy’. Instead of using the 10 MHz most widely used in ophthalmic diagnostic ultrasound, UBM uses ultrasound frequencies in the 50 to 100 MHz range, allowing examination of living subsurface ocular tissues at very high resolution. UBM has found widespread usage as a method of imaging much ocular pathology, from adnexal, conjunctiva, scleral, corneal, anterior chamber to anterior vitreous and retina. However, its major contribution has been to the understanding of the structure of the anterior segment, particularly in glaucoma.

## INSTRUMENTATION: ULTRASOUND BIOMICROSCOPY

**Fig. 1:**
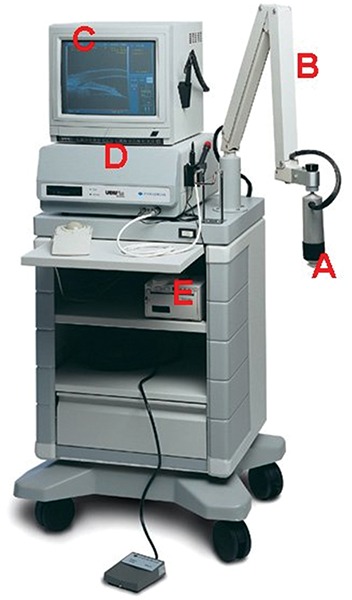
The current UBM―P45 workstation (Paradigm Medical Industries, UT, USA). A: Ultrasound transducer and probe; B: Articulated arm; C: Computer monitor; D: Main processing unit; E: Printer

The first clinical model and prototype was developed in the late 1980s, and the first clinical images were taken in March 1990. In cooperation with Pavlin, Zeiss-Humphrey Inc. (San Leandro, CA, USA) developed the first commercial model (Model 840) of the UBM in 1994. Recently, the product line was sold to Paradigm Inc. Additional software has been developed subsequently for image analysis. The newest model (Fig. 1) is the P60 workstation (Paradigm Ins. US).

**Fig. 2:**
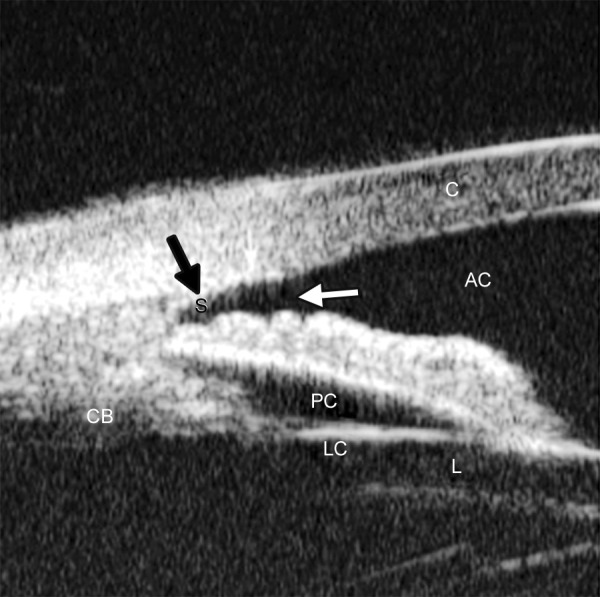
Illustration of major anatomical landmarks in UBM images. (C: Cornea; AC: Anterior chamber; S: Scleral spur; CB: Ciliary body; PC: Posterior chamber; LC: Lens capsule; L: Lens). The black arrow shows the most important landmark for drainage angle measurement― scleral spur

The development of UBM equipment was made possible by advances in transducer, high-frequency signal processing and precise motion control technology. The principal components of UBM are shown in Figure 1. The transducer is the critical component. By moving a transducer linearly over a 5 mm image field, sonographic data are generated along each of 512 lines (8 micron between lines) (Fig. 2). The signal is amplified in proportion to the depth from which it originated using so called ‘time-gain compensation’. After signal processing, ultrasound data can be converted from analog to digital format and transferred to a high speed scan converter, and eventually displayed on a video computer. Only the signals returning from a 5 × 5 mm area centered at the focal depth are stored. In prototype models, a frequency range of 50 to 80 MHz with a field of view of 4 × 4 mm was selected because it can give a useful compromise that allows all the important structures of the anterior segment to be visualized.

According to the principles of ultrasound physics, image quality is dependent on the frequency of the ultrasound, the ratio of the focal length to the transducer diameter (f-number) and the length of the pulse. Higher frequency and shorter focal length are usually associated with higher resolution of the images but poorer penetration. For example, a device with a high frequency (80 MHz) and short focal length (1.2 mm) can give a very sharp image of the cornea showing the epithelial layer at 50 microns resolution, although the deeper structures of the cornea are not shown clearly.

Measurement accuracy of the imaging system is dependent on the lateral and axial resolution, the stability of mechanical motion and the pixel size of the image. The lateral resolution (transverse to the direction of pulse propagation) depends on the distribution of ultrasound in the field of the transducer, which has a width at half maximum given by the product of the wavelength and the f-number. Therefore, a transducer of 80 MHz and f 2.2 has poorer resolution than 80 MHz/f 1.2. The 80 MHz/f 2.2 can capture the resolution of 50 microns. The axial accuracy (resolution) is determined by the speed of sound in the various tissues, for example, 1542 m/s in the iris to 1620 m/s in the sclera. There are two terms describing the axial accuracy: ‘Instrument axial resolution’ and ‘measurement precision’. Instrument axial resolution is the instrument’s capability to distinguish two surfaces when they are brought closer and closer together. ‘Measurement precision’ can be significantly better than axial resolution in some special conditions, such as when the two planar interfaces are well resolved and parallel, e.g. the anterior and posterior surfaces of the cornea (Pavlin and Foster 1995).

## EXAMINATION TECHNIQUE: ULTRASOUND BIOMICROSCOPY

The UBM examination technique is similar to B-mode ultrasound. Transducer direction and manipulation of the probe is guided by looking at the image on the screen. Major differences include an oscillating probe without a covering, the use of a water bath and the finer movements required.

The patient is examined in a supine position facing the ceiling. After topical anesthesia, a specially-designed eyecup (22 to 24 mm diameter) is used to separate the eyelids and form a water bath environment. This is filled with a viscous, sono-lucent coupling fluid such as methylcellulose (1-2.5%). Some examiners use normal saline to fill the cup after sealing the interface between the eye and the base of the cup with 2.5% methylcellulose.

Images are stored in an electronic format on a computer attached to the device. This format UBM image files are not compatible with commercially available image editing software. Patient’s name, ID number, date of examination and laterality of eye are stored in a separate file. Reviewing images and the derivation of measurements from the images has to be done on the UBM’s computer unit or a PC which uses suitable software to process and display the images.

## QUANTITATIVE MEASUREMENT OF UBM IN ANGLE ASSESSMENT

UBM has proved to be a great asset in the study of angle-closure. Radially-orientated images through the limbus provide a cross-sectional view of the anterior chamber angle. The corneoscleral junction and scleral spur can be distinguished in the majority of cases. The scleral spur is usually clearly visible. Quantitative analysis of angle anatomy depends on its accurate localization. However, in most instances, UBM examination is used for qualitative analysis, such as a confirmation of the angle appositional closure, existence of ciliary rotation or to identify other abnormalities of the ciliary body and angle. Quantitative analysis of the geometric angle width is usually only employed as a research tool.

The quantitative analysis of UBM images in the study of angle-closure usually addresses three specific issues: Quantifying the angle width by measurement of either linear distance or geometric angle, and the measurement of area between iris and trabecular meshwork.

Additional measurements of iris thickness and contour as well as the relationship between iris and ciliary body may also be made.

### Angle Width Quantified by Linear Distance

**Fig. 3:**
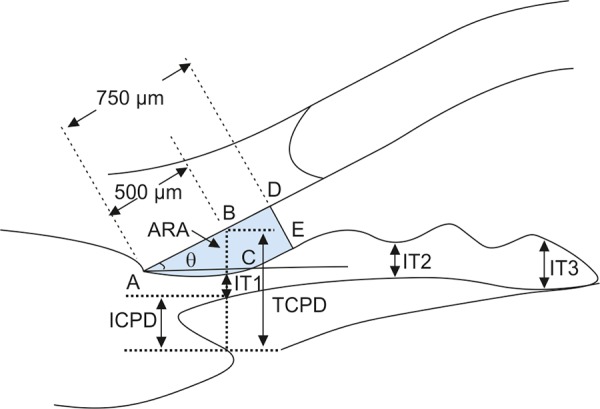
Parameters commonly used for image analysis of UBM. ARA: Angle recess area at 750 microns anterior to the scleral spur; IT1-3: Iris thickness at various locations to the scleral spur; TCPD: Trabecular ciliary process distance; ICPD: Iris ciliary process distance

As previously discussed, gonioscopic grading depends on the examiner’s experience and subjective judgment. The development by Pavlin’s group of a UBM method of quantifying angle width in degrees has realized the hopes of many for such a technique (Pavlin, Harasiewicz, et al 1992). The most commonly used index of angle width is the angle-opening distance (AOD). The scleral spur is identified and a point on the internal wall of the corneoscleral plane at a given distance from the scleral spur (most often either 250 or 500 microns) is identified. From this point anterior to the scleral spur, a line perpendicular to the plane of trabecular surface is extended to meet the surface of the iris (Fig. 3). The length of this line gives the AOD, termed AOD 250, 500 or 750, dependent on the distance from the scleral spur. AOD at 500 microns was reported to be 347 ± 181 microns in normal eyes (Pavlin, Harasiewicz, et al 1992).

### Angle Width by Degree of Angle

Measurement of the angle in degrees (trabecular-iris angle, TIA81) was also proposed by Pavlin, defining the angle as the apex of lines passing through the point on the meshwork 500 microns anterior to the scleral spur and the point on the iris perpendicularly opposite. However, measurement of the angle using this method was problematic and felt to be of limited validity because of the irregular contour of the iris (see Fig. 3) (Pavlin, Harasiewicz, et al 1992).

### Angle Width by Angle Recess Area

Ritch’s group in New York further refined this measurement of the anterior chamber angle. Realizing that all variations in angle anatomy cannot be summarized by either a single measure linear distance or geometric angle because of variations in the contour of the peripheral iris which contribute to the risk of angle closure, Ishikawa developed a parameter called the ‘angle recess area (ARA)’ (Ishikawa, Esaki, et al 1999). The ARA was defined as the area bordered by the anterior iris surface, corneal endothelium and a line perpendicular to the plane of the corneal endothelium drawn to the iris surface from a point 750 microns anterior to the scleral spur (see Fig. 3).

### Measurement of Iris Contour, Thickness and Relationship with Ciliary Body

The relationship between the iris and the trabecular meshwork is central to the understanding of angle-closure. Clearly, variation in thickness and shape of the iris are major variables determining the nature of this relationship. Attempts have been made to measure iris contour and thickness by UBM. In the study of pigment dispersion syndrome, Potash described an index of iris concavity (Potash, Tello, et al 1994). A line was first extended from the most peripheral point to the most central point of the iris pigment epithelium. A perpendicular line is created from this line to the iris pigment epithelium at the point of the greatest concavity or convexity. Pavlin et al proposed that iris thickness measured at three locations, perpendicular to the horizontal plane of the iris, the measurements of thickness were made at 500 microns from the scleral spur (IT1), at 2 mm from the iris root (IT2), and at the maximum iris thickness near the pupil margin (IT3) (see Fig. 3).

**Table 1:** **Table 1:** Parameters proposed by Pavlin and Foster

*Parameters*		*Description*	
AOD		Distance between the trabecular meshwork and the iris at 500 microns anterior to the scleral spur	
TCPD		Distance between the trabecular meshwork and the ciliary process at 500 microns anterior to the scleral spur	
IT1		Iris thickness at 500 microns anterior to the scleral spur	
IT2		Iris thickness at 2 mm from the iris root	
IT3		Maximum iris thickness near the pupil margin	
ICPD		Distance between the iris and ciliary process along the line of TCPD	
ILCD		Contact between the iris and the lens	
TIA91		Angle of the angle recess	

In order to describe the location of the ciliary processes, Pavlin used the trabecular meshwork ciliary process distance (TCPD), measuring this perpendicularly through the iris to opposing body of the ciliary process from a point 500 microns anterior to the scleral spur along the plane of the corneal endothelium (see Fig. 3).

Iris-lens contact distance (ILCD) is another measurement believed to give a measure of pupil block. It is measured along the iris pigment epithelium from the pupil border to the point where the iris physically leaves contact with the anterior surface of the lens (see Fig. 3).

Ishikawa reviewed and illustrated most of these UBM measurements proposed by Pavlin recently as seen in Table 1.

Variation in the UBM measurement depends on the image acquisition, image analysis and physiological variability of angle structures. Variation at the stage of image acquisition occurs mainly as a result of inconsistencies in alignment, failure to control accommodation and room illumination. Direction of gaze can be standardized by placing five markers on the ceiling to optimize orientation of the eye when measuring different quadrants (Urbak, Pedersen, et al 1998). However, this method is too time-consuming for use in clinical practice. Standardization of other sources of error is more difficult to achieve.

In order to assess the reproducibility in the quantitative examination using the UBM, Tello calculated interobserver and intraobserver variation in measurements (Tello, Liebmann, et al 1994). Tello compared image analysis by three observers repeatedly measuring the same four UBM images. Intraobserver agreement was good in central cornea thickness and anterior chamber depth (coefficients of variation, CV < 3.8%) but poorer in angle measurements (CV 1.3%). Interobserver agreement in general was poor and varied considerably, and was thought to be affected by subjective interpretation of location of anatomical landmarks. The TCPD and ID1-3 were less good, while the measurements of AOD and angle width in degrees were the worst.

The authors used ‘coefficient of variation’ (CV) (standard deviation divided by mean) to assess agreement of measurements. However, this index may not be the most appropriate because it assumes the data have a Gaussian distribution. Furthermore, it is probably not appropriate to use this parameter to evaluate the variation in just three paired observations. Also, the study suffered from a small number of subjects who all had normal, wide angles, and as a result did not test variation across the entire range of possible values. This limits the extrapolation of the results to eyes with narrow angles which may be more difficult to assess, because of crowding of anatomical landmarks.

**Fig. 4:**
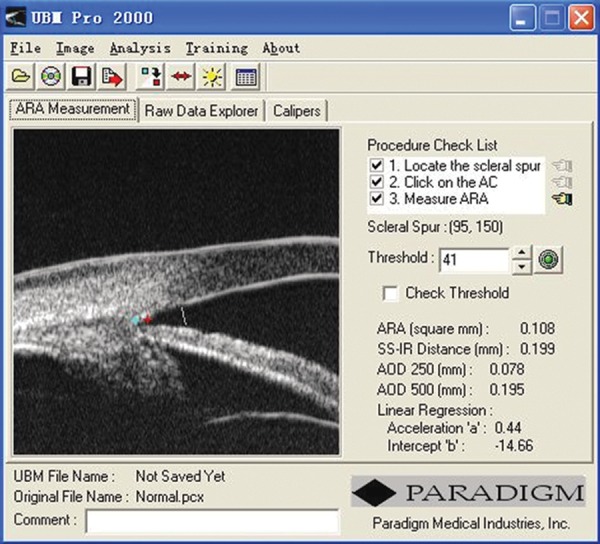
A screen image from the UBM Pro 2000 software package

Urbak performed a similar study on 50 UBM images obtained by three observers. The angle width in these images was not stated in the paper. Using the same statistical methods, the conclusions were similar (Urbak, Pedersen, et al 1998). Spaeth carried out a more careful evaluation of assessment of angle configuration by UBM (Spaeth, Azuara-Blanco, et al 1997). After enrolling 22 patients from his glaucoma clinic, many of them are angle-closure patients, he attempted to classify the images by iris insertion, angular width and iris profile, using the same scheme as his (Spaeth’s) gonioscopic grading system. Two observers graded the UBM images independently. The intraobserver agreement overall was good with kappa value ranging from 0.83 to 0.92 for the three angle characteristics. The interobserver agreement was slightly lower for iris insertion (k = 0.79) and iris curvature (k = 0.84), but measurements of angular width showed remarkably good agreement (k = 0.95). This study is the first to demonstrate good intraobserver and interobserver agreement for UBM images analysis. It also had the advantage that the sample represented a wide range of angle configurations.

It was commonly pointed out that different observers may choose different reference points as the ‘landmark’ for the location of scleral spur and trabecular meshwork outline. There is also the difficulty in measuring exact distances on UBM images. In an attempt to minimize measurement variability, Ishikawa and Ritch developed computer software which calculated AOD and ARA automatically once the scleral spur is identified. This software, UBM Pro 2000, has been made commercially (Paradigm Co) (Ishikawa, Liebmann, et al 2000). A new feature was incorporated in the commercial version of this software, which allows AOD to be calculated at different distances from the scleral spur. Additionally, a regression line is constructed to reflect the iris profile at distances from 250 to 750 microns anterior to the scleral spur (Fig. 4).

UBM provides a unique tool that helps to describe the *in vivo* cross-sectional morphological characteristics of the anterior segment. It can delineate the drainage angle, cross-sectional iris profile, ciliary body, iris insertion, and zone of iris-lens contact, most of which are not visible when using any other ocular biometric devices. In clinical practice, it may help to identify plateau iris, iris crowding and also perhaps longitudinal change of the drainage angle. Limited evidences suggested it can also be used to examine the equatorial zonular region (Pavlin, Buys, et al 1998).

## CLINICAL APPLICATION OF ULTRASOUND BIOMICROSCOPY *CORNEA*

### Normal Cornea (Fig. 5)

UBM is able to differentiate the cross-sectional structure of cornea. Epithelium forms a smooth reflection line on the surface. The highly reflective line right below the epithelium is Bowman’s membrane. The distance between the smooth surface line and highly deflective line suggests the thickness of corneal epithelium. Corneal stroma is the layer with lower and regular reflection. The 3rd high reflective line is the Descemet’s membrane and endothelial layer. These two layers are usually difficult to differentiate in UBM imaging.

### Pathology of the Cornea

Corneal Edema (Fig. 6)

The typical features are irregular surface (epithelium) and increasing reflectivity of corneal stroma. Bulla may be presented on anterior surface line in some severe cases.

Blood Staining of the Cornea

*Case presentation:* A 17-year-old woman presented with eye contusion for 1 week. Examination revealed a visual acuity of light perception, intraocular pressure 45 mm Hg (noncontact tonometry). Slit lamp examination was notable for corneal edema, brown stain of corneal stroma, anterior chamber fully filled by blood, other structures being not visible. UBM findings are shown in Figures 7 and 8.

*Clinical implication from UBM:* Initially, iris, lens and angle were not visible using either slit lamp or B-ultrasound, UBM examination is able to detect these changes that are important for further clinical management.

**Fig. 5:**
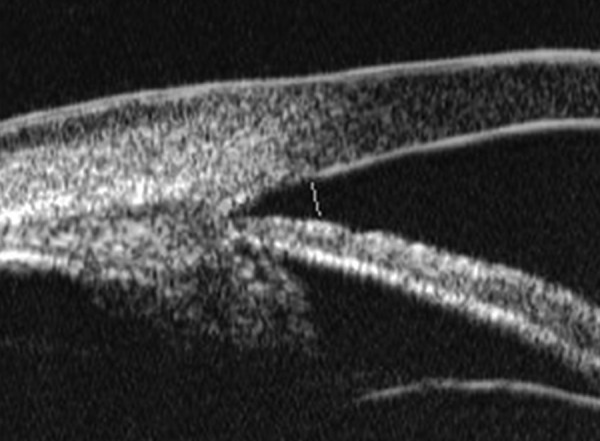
Normal cornea

**Fig. 6:**
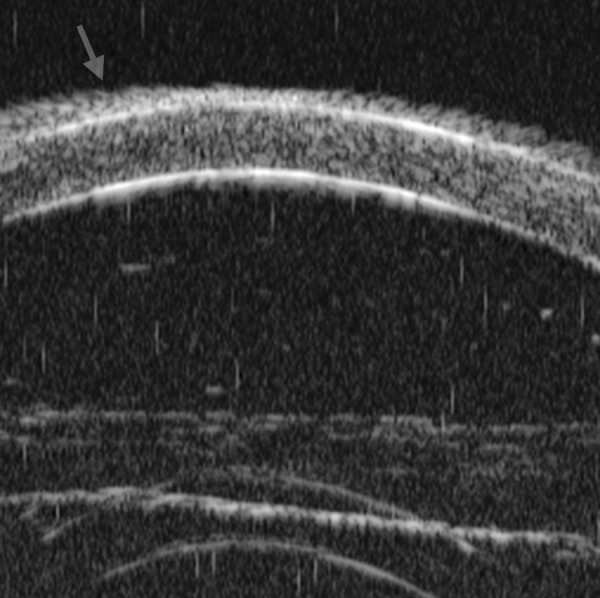
Corneal edema. The epithelium layer is thickened loosing smooth and regular surface (white arrow). The stroma also turns thickened appearing highly reflective

**Fig. 7:**
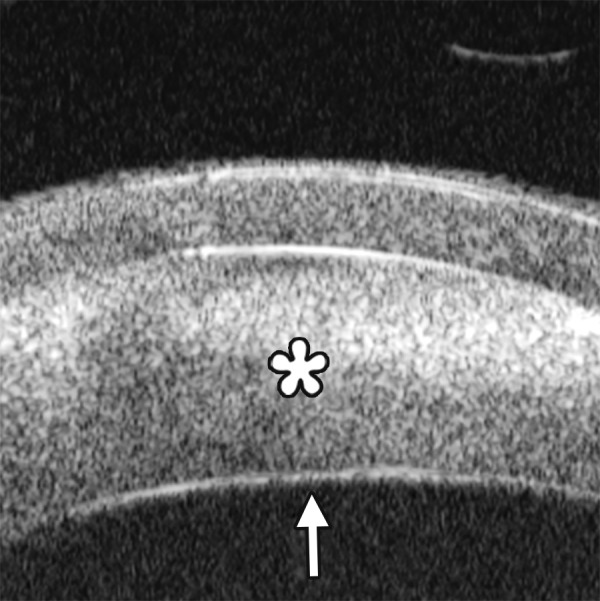
UBM image of blood staining of cornea, showing epithelial surface edema, high reflection in stroma, anterior chamber shallowing with suspected lens dislocation (the highly reflective line marked by white arrow represents anterior surface of lens); anterior chamber was filled with blood clot (asterisk) of various densities

**Fig. 8:**
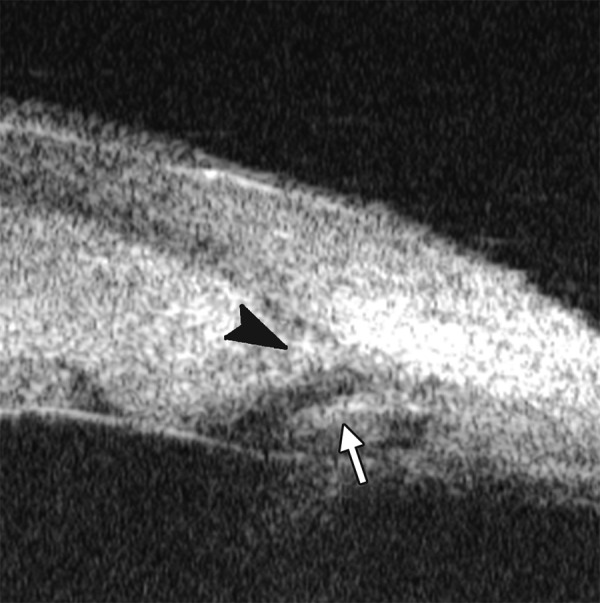
Angle changes in the same patient. The angle structure is illustrated: Iris is visible (white arrow), blood clot was presented in the peripheral anterior chamber (black arrowhead)

**Fig. 9:**
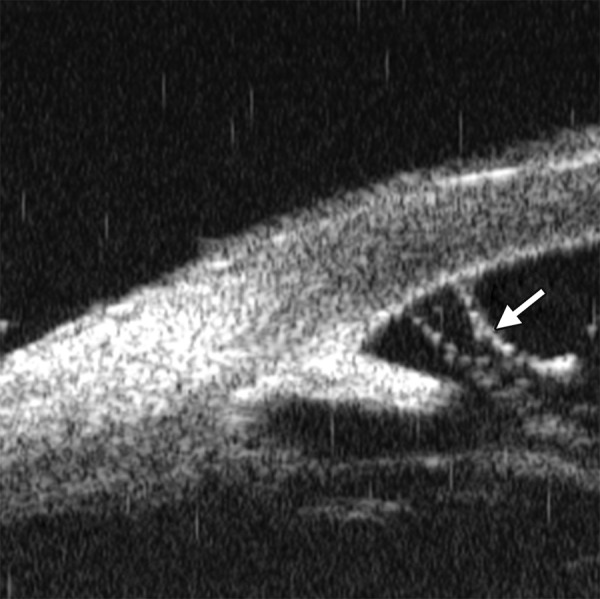
Descemet’s membrane detachment after phacosurgery. The detached Descemet’s membrane is marked by white arrow as highly reflective line from posterior corneal surface near the incision site

**Fig. 10:**
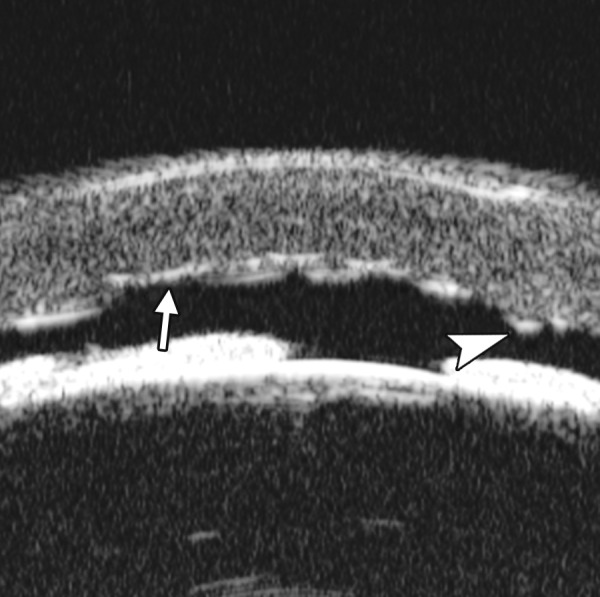
Endothelial changes in endothelial keratitis. Corneal stroma is thickened with irregular endothelium surface (white arrow). KP is illustrated as hyperechoic spots attached on the corneal back (white arrowhead). Furthermore, the examination demonstrated shallow ACD and iris atrophy in this case

Descemet’s Membrane Detachment (Fig. 9)

*Case presentation:* A 56-year-old man presented with persistent corneal edema 4 days after phacosurgery. UBM imaging revealed a thin membrane detached from the back of cornea. Corneal edema was also present.

*Clinical implication:* Descemet’s membrane is normally a thin layer of transparent tissue, which is hardly able to identify by routine slit lamp when it is presented with coexisting corneal edema. UBM is able to identify the detachment and describe the location and extent of this detachment.

Descemet’s Membrane Folds

*Case summary:* A 23-year-old man was presented complaining of decreased vision, pain, photophobia for 2 days. Examination revealed visual acuity as 0.2, IOP 29 mm Hg. The preliminary diagnosis was endothelial keratitis (Fig. 10) with possible trabecular meshwork damage. Slit lamp examination demonstrated corneal edema, Descemet’s membrane folds. UBM demonstrated similar changes as what were able to observe by slit lamp. In cases with severe corneal edema, UBM is more useful identifying the changes in corneal endothelium and trabecular meshwork.

**Fig. 11:**
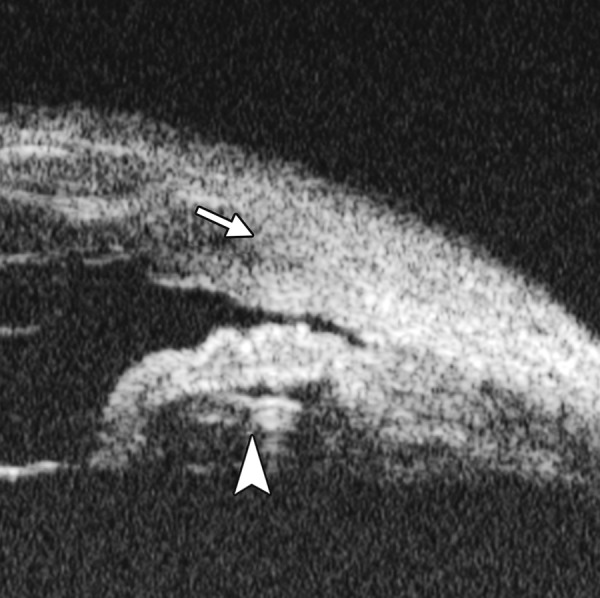
Corneal leukoma. UBM image shows obviously thickened cornea with high reflection (white arrow). The proliferative tissues beneath iris (white arrowhead) pushed the root upward leading the angle nearly closed, which may attributable to the secondary glaucoma and iris-lens synechiae

Corneal Leukoma

*Case presentation:* A 45-year-old male was diagnosed as herpes simplex keratitis with leukoma formation. Visual acuity was hand motion. IOP was 35 mm Hg. Slit lamp found corneal opacity throughout cornea, localized thinning, only peripheral chamber partially visible, other structures of anterior chamber and iris being not visible. UBM revealed increasing reflectivity in corneal stroma, several localized areas of thinning of the cornea, cornea deformity and normal anterior chamber structures.

*Clinical implication:* UBM is able to demonstrate anterior chamber structure and identify existence of anterior synechiae. These features are not clearly visible in routine slit lamp examination.

**Fig. 12:**
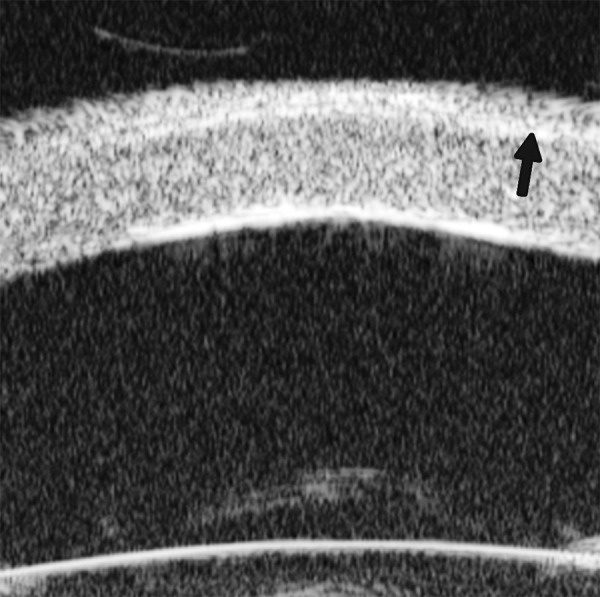
UBM demonstrated Bowman’s membrane loses its continuity and turned irregular with localized hyperechoic dots and flacks (black arrow). Corneal stroma appears edema because of epithelium dysfunction

**Fig. 13:**
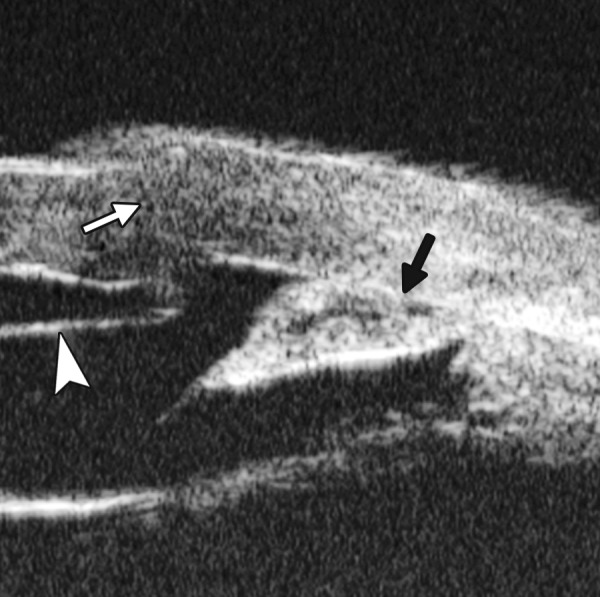
UBM image shows secondary angle closure due to severe PAS (black arrow), the irregular graft-host conjunction of penetrating keratoplasty (white arrow), as well the detached Descemet’s membrane of implanted cornea, which may result in its edema

**Fig. 14:**
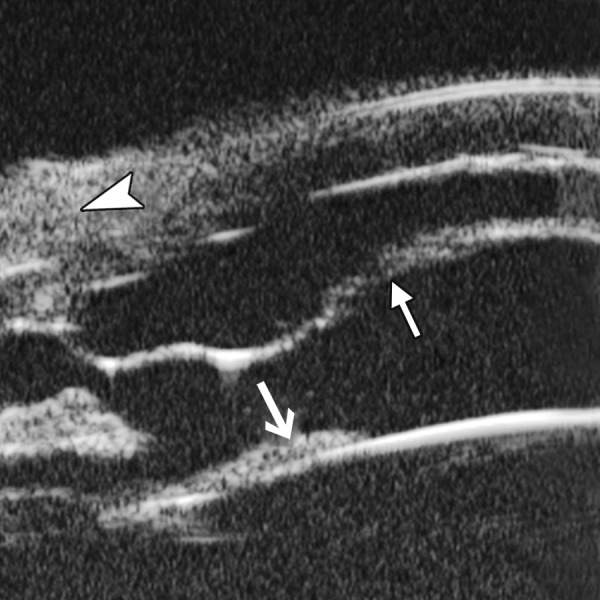
UBM image shows separation of host Descemet’s membrane (thick white arrow) and implanted cornea, the graft-host conjunction (white arrowhead) and the fibrous membrane attached on the anterior lens surface (slender white arrow)

Corneal Dystrophy

*Case summary:* A 19-year-old man was complaining of pain, foreign body sensation and decreased vision for 1 week. The primary diagnosis was granular stromal dystrophy. Slit lamp examination revealed corneal cloudy in the corneal stroma, localized grey color granular lesions in the Bowman’s membrane were identified. UBM findings are shown in Figure 12.

## SURGERY OF CORNEA

### Postoperative Complications

Secondary Glaucoma

*Case summary:* A 32-year-old female presented with IOP elevation (35 mm Hg) at 1 month after partial penetrating keratoplasty. UBM imaging demonstrated that iris incarceration in the graft-host junction at 3 to 9 clock hours. Anterior synechiae resulted from this iris anterior synechiae could eventually lead to secondary glaucoma. Furthermore, UBM is useful for visualizing the aberration at graft-host junction and anterior drainage angle in greater details when the visualization is usually compromised by scarred or edematous cornea (Fig. 13).

Interlamellar Liquid in Lamellar Keratoplasty

*Case summary:* A 40-year-old female, deep lamellar keratoplasty was performed because of corneal leukoma. At day 3 postoperatively, the donor cornea was severe edema with no underlined structure visible. UBM demonstrated the existence of liquid between donor and host cornea, suggesting potential perforation of Descemet’s membrane of the host cornea (Fig. 14).

## SCLERA

The space for examination is limited by the lid fissure width and eyecup. UBM is only able to examine the area anterior to equator. Normal sclera is a dense connective tissue. Typical UBM feature is a regular high reflectivity signals (sclera) with relatively lower reflective tissue surrounding or inside, e.g. episcleral tissue, ciliary body or choroids. Thus, UBM is commonly used to differentiate structural abnormalities of sclera or episclera tissues, it is also useful for the assessment of treatment outcomes.

### Episcleritis

Case Summary

**Fig. 15:**
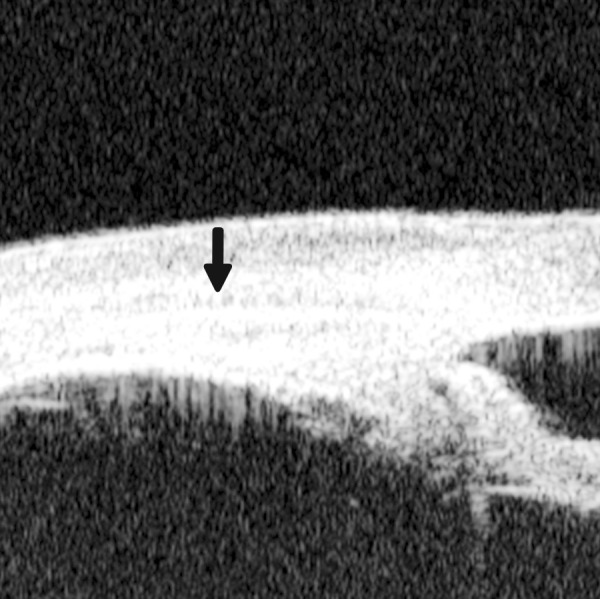
Episcleritis. UBM reveals thickening of the episclera tissues, but the stroma of sclera is not affected. A distinct border (black arrow) was observed between scleral stroma and episcleral tissues (including bulbar conjunctiva and Tenon’s capsule)

A 45-year-old female presented complaining of redness and pain in the right eye for 1 week. Slit lamp revealed a localized injection of the bulbar conjunctiva, hyperemia of episclera, mild palpation pain. UBM examination results are shown in Figure 15.

### Scleritis

Case Summary

A 57-year-old female presented with pain, redness and photophobia for 2 weeks. History included a rheumatologic disorder. IOP was 18 mm Hg. Slit lamp revealed slight edema of cornea, injection of bulbar conjunctiva. Palpation pain was apparent.

**Fig. 16:**
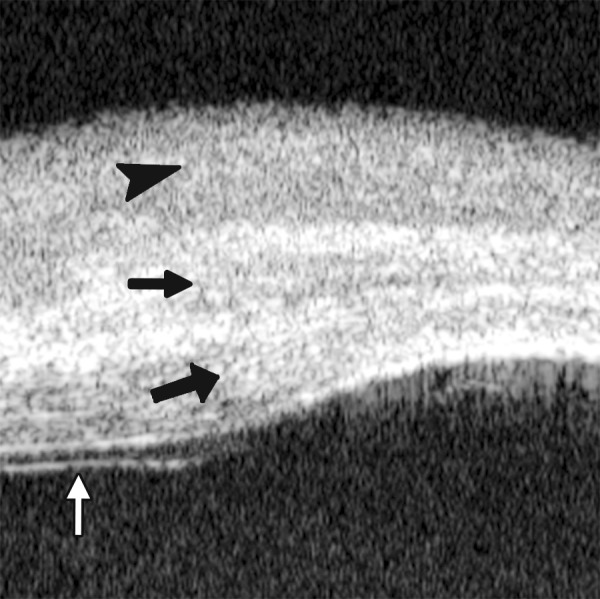
UBM imaging demonstrates the inflammation affects both episclera and sclera stroma―thickening of episclera (top black arrowhead) and diffuse decreasing reflectivity regions within the stroma (middle black arrow). Internal layer of sclera is also affected (bottom black arrow). Furthermore, this case was complicated with retinal detachment (white arrow)

This case was diagnosed as posterior scleritis. Usually, the disease is easily to be misdiagnosed for without obvious specific objective signs in the anterior ocular segment except congestion. UBM is useful to detect inflammatory damage of the sclera as well as close tissues, such as uvea and retina (Fig. 16).

### Limbal Staphyloma

Case Summary

**Fig. 17:**
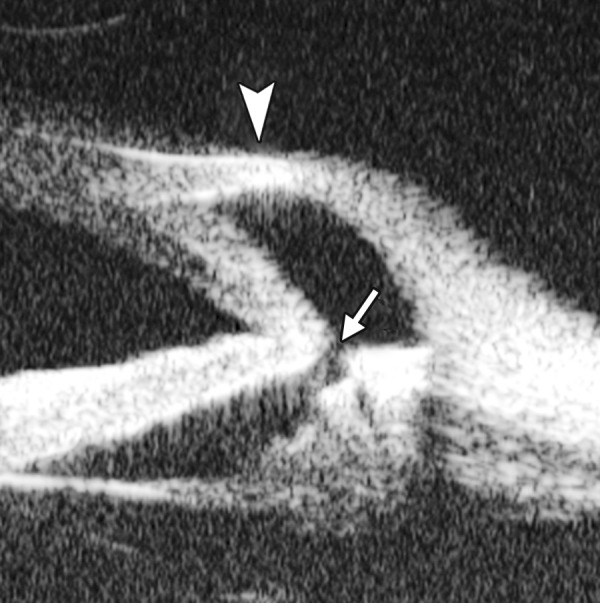
The UBM image shows the bulging of the limbal wall (white arrowhead) contains hypoechoic fluid rather than tissue. There is a pathway connected to the posterior chamber (white arrow)

A 30-year-old female, with a history of recurrent anterior scleritis, complained of darkly colored lump in the limbal area of the right eye for 3 years. Preliminary diagnosis was limbal melanoma. Slit lamp examination found a 4 × 3 mm lump on the limbus at 8 clock hour. UBM findings included the thinning of sclera, loss of ciliary body and a space underneath the sclera with fluid and connecting to anterior chamber (Fig. 17). UBM result excluded the melanoma diagnosis and confirm the diagnosis of staphyloma in reference to the recurrent scleritis history.

**Fig. 18:**
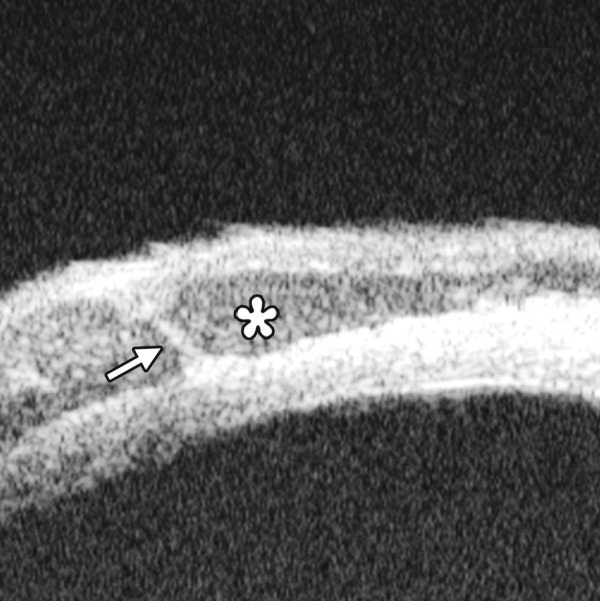
The hypoechoic area underneath superficial sclera (asterisk) is the abnormal vessels. White arrow refers to the vessel wall

**Fig. 19:**
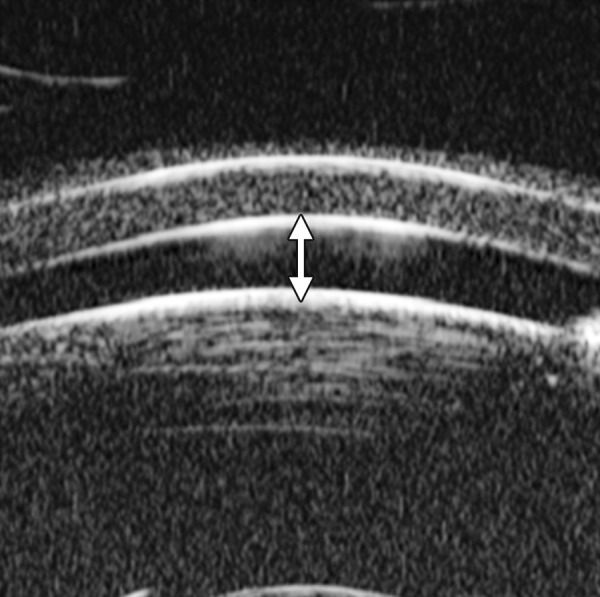
UBM image shows the axial ACD is equal to the central corneal thickness (white arrow)

**Fig. 20:**
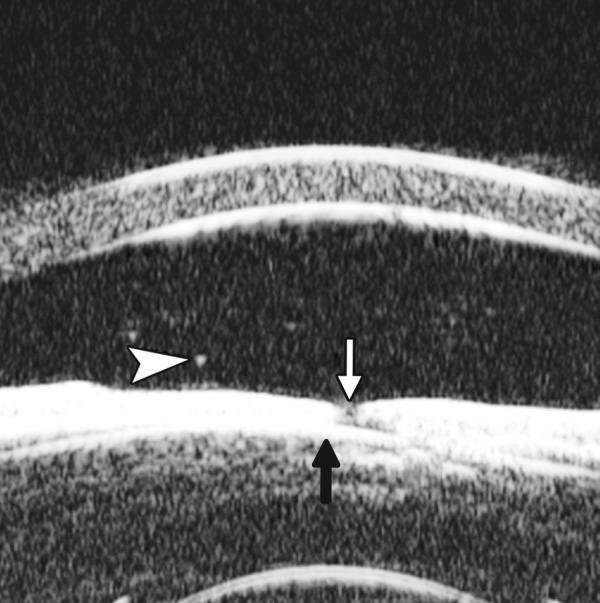
UBM demonstrated floater in anterior chamber (white arrowhead), fibrous membrane covered pupil region (white arrow). Posterior synechiae was formed between iris and lens in the pupil region (black arrow). Anterior lens cortex was opaque

**Fig. 21:**
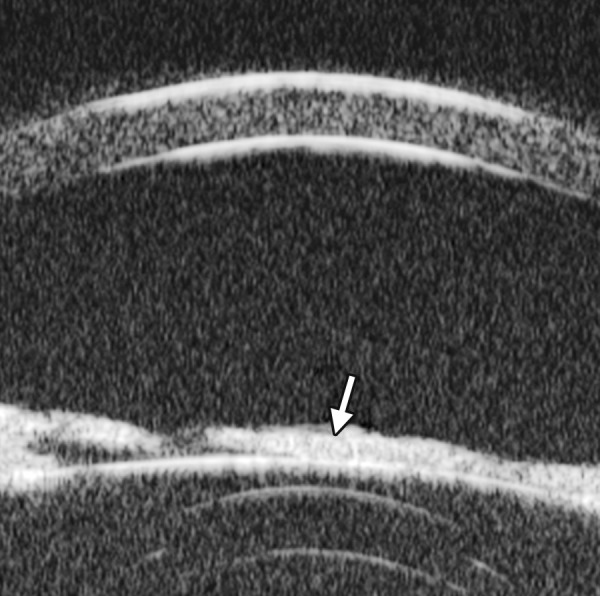
Iris tissue over the lens

**Figs 22A and B:**
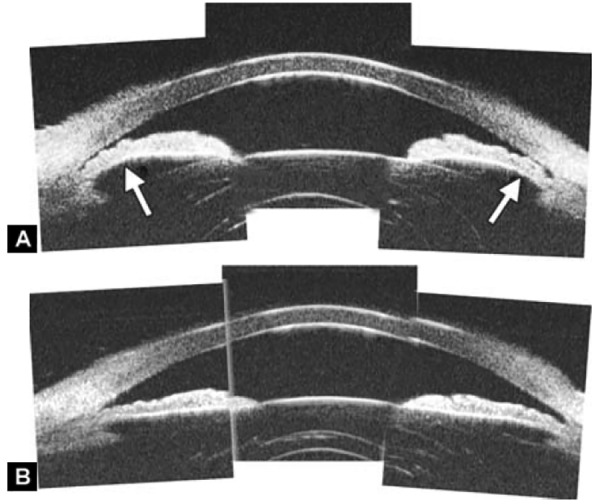
This UBM image-montage shows the effect of laser iridotomy. Angles have opened after laser PI, and the bombe iris (marked by white arrow in Fig. A) becomes flat (shown as Fig. B). The iris insertion is located in the middle of the anterior face of the ciliary body, suggesting the location of iris insertion is important

### Scleral Vascular Abnormality

Case Summary

A 65-year-old man was diagnosed as Sturge-Weber syndrome with diffuse retinal detachment. Visual acuity was hand motion and IOP was 24 mm Hg. UBM identified an enlargement of vessel inside the scleral stroma on the nasal superior quadrant.

## ANTERIOR CHAMBER AND ANTERIOR CHAMBER ANGLE

### Anterior Chamber

Shallow Anterior Chamber

*Case summary:* A 55-year-old female suffered from over filtration after trabeculectomy. The central anterior chamber depth on slit lamp was approximately 1 CT.

Pupillary Occlusion

*Case summary:* A 35-year-old female was diagnosed as anterior uveitis for 1 year. Visual acuity was hand motion and IOP was 21 mm Hg. Slit lamp revealed fibrous exudation in the pupil region and the pupil was completely occluded.

Congenital Persistent Pupillary Membrane

*Case summary:* A 9-year-old boy was diagnosed as persistent pupillary membrane. The diagnosis was verified by slit lamp and the observation of fundus was obscured. UBM showed a piece of residual membrane covering the entire pupil region (white arrow), there was a close contact between the membrane and lens anterior capsule (probably synechiae). The existence of synechiae suggested YAG laser alone may not able to remove the membrane, attention should be paid not injury the lens if surgery is planned.

### Anterior Chamber Angle: Primary Angle-Closure Glaucoma

*Pupil Block Predominated* Before laser PI (Fig. 22A) After laser PI (Fig. 22B)

Non-pupil Block

**Figs 23A and B:**
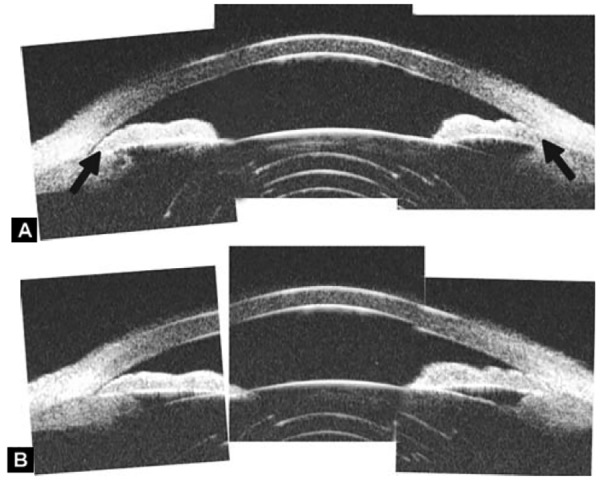
Typical anteriorly-rotated, large ciliary body supports the peripheral iris (black arrow in Fig. A), preventing the peripheral iris moving backward after laser iridotomy (shown in Fig. B). Anterior location of the iris insertion also plays a role in this mechanism

Before laser PI (Fig. 23A) After laser PI (Fig. 23B)

Anterior rotation of ciliary body, anterior iris insertion, iris thickness and configuration at insertion, all contribute to the angle-closure in those predominantly caused by non-pupil block mechanism. Laser peripheral iridotomy only has limited efficacy in these cases. Argon laser peripheral iridoplasty may have better outcome but need further evidence to prove.

Peripheral Anterior Synechiae (PAS)

**Figs 24A and B:**
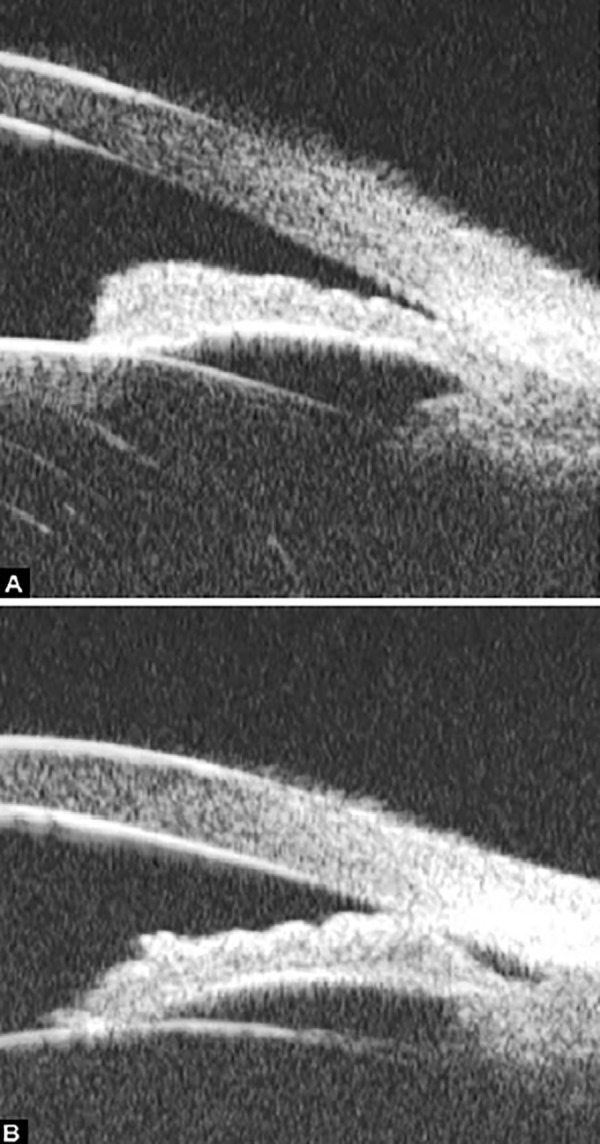
PAS of B-type and S-type (these images demonstrate appositional closure instead of PAS)

PAS is a permanent adhesion between the peripheral iris and trabecular meshwork (or the back of cornea). This is believed to be the anatomical basis for chronic angle closure. The mechanism of the establishment of PAS is not entirely understood. Gorin described the gonioscopic features of the drainage angles and postulated two kinds of closure: B-type (closure from the bottom of the drainage angle, as marked by black arrow in Figure 24A) and S-type (closure that starts from Schwalbe’s line, as marked by white arrow in Figure 24B).

PAS is also able to extend from a localized one to all over the entire circumference of the drainage angle. Routine UBM usually is not able to differentiate PAS with appositional closure, but UBM on dynamic changes may help differentiate in some but not all cases.

### Dark Room Provocative Test

Provocative tests are intended to stimulate the physiological conditions under which angle-closure may occur. The outcome measurement, rising of IOP, is used to differentiate those with increasing risk and require further intervention. Observation on the angle-closure after provocative test using gonioscopy could be challenging as the illumination and gonioscopy manipulation will probably open the angle. UBM can be performed in dark room and involves relatively less manipulation, thus, it is better than gonioscopy to observe the angle-closure after provocative test.

The angle is obviously widened with pupil construction under light stimulation. This test can be used to identify an appositional closed angle which is important to guide the following therapy.

**Figs 25A and B:**
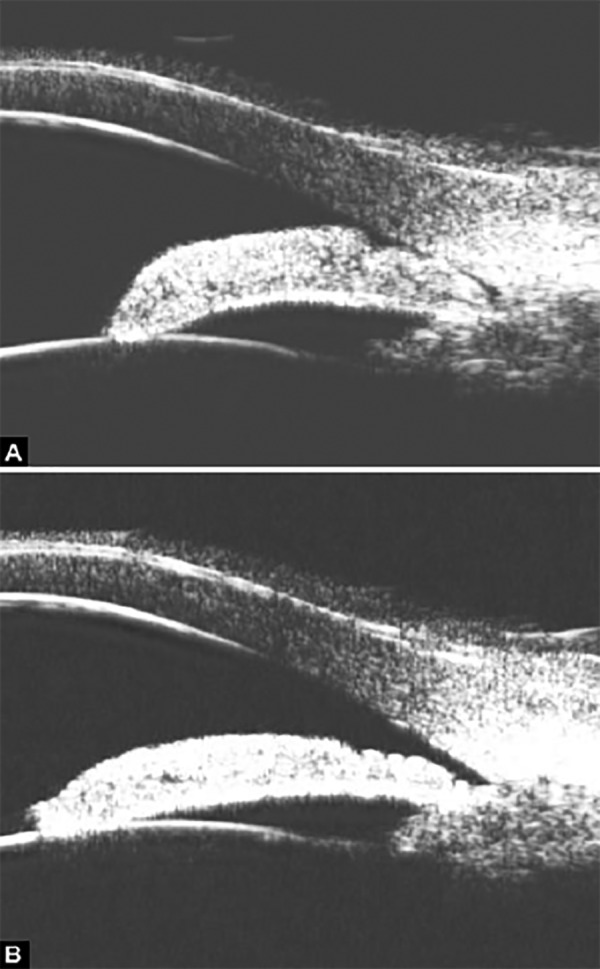
(A) UBM drainage angle in dark and (B) light

### Secondary Angle Closure

1. Angle closure secondary to iris abnormalities (see other sections)
2. Secondary to lens subluxation (see other section)
3. Neovascular glaucoma.

Neovascular glaucoma is usually secondary to retinal ischemic abnormalities, e.g. central retinal vein occlusion, diabetic retinopathy and Eales’ diseases. Usually, IOP elevation typically results from occlusion of the drainage angle by fibrovascular membrane.

**Figs 26A and B:**
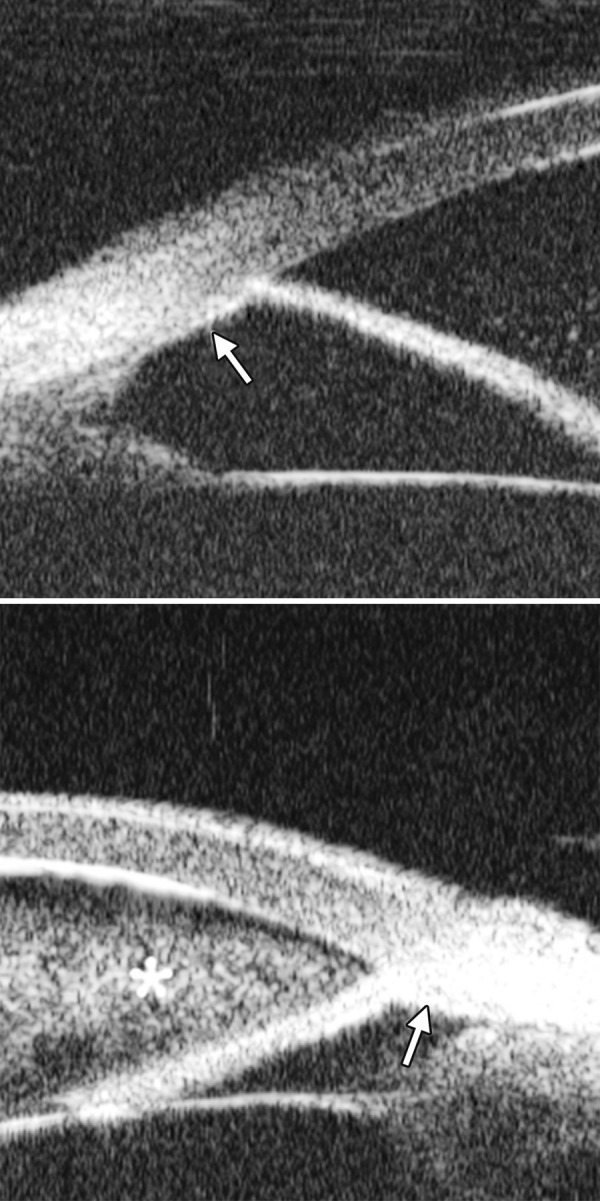
Neovascular glaucoma

*Case summary:* A 58-year-old female has been diagnosed with diabetic retinopathy complicated with multiple recurrent vitreous hemorrhage for 7 years. Panretinal photocoagulation has been performed before. Visual acuity was 0.01, IOP was 45 mm Hg. Slit lamp examination confirmed an edematous cornea and irregular, nonradial vessels were presented in the iris stroma. Peripheral anterior chamber was absent. UBM examination revealed the atrophy of iris stroma, anterior synechiae secondary to the contraction of the fibrovascular membrane. The drainage angle was completely closed (white arrow in Fig. 26A). Figure 26B demonstrated the hyphema resulting from the breaking of neovessels (asterisk). In these particular cases with corneal edema or hyphema, UBM is very useful to identify the changes of iris and angles (white arrow in Fig. 26B).

4.    Iridocorneal endothelial syndrome (ICE, see other sections)
5.    Pigmentary dispersion syndrome (see other sections)
6.    Malignant glaucoma.

It was believed that the blockage of the aqueous flow at the level of ciliary body or anterior vitreous face causing malignant glaucoma. The aqueous humor is misdirected into vitreous cavity producing expansion of vitreous cavity and increase of posterior segment pressure. Iris-lens diaphragm was forced to move forward by the high vitreous pressure.

**Figs 27A and B:**
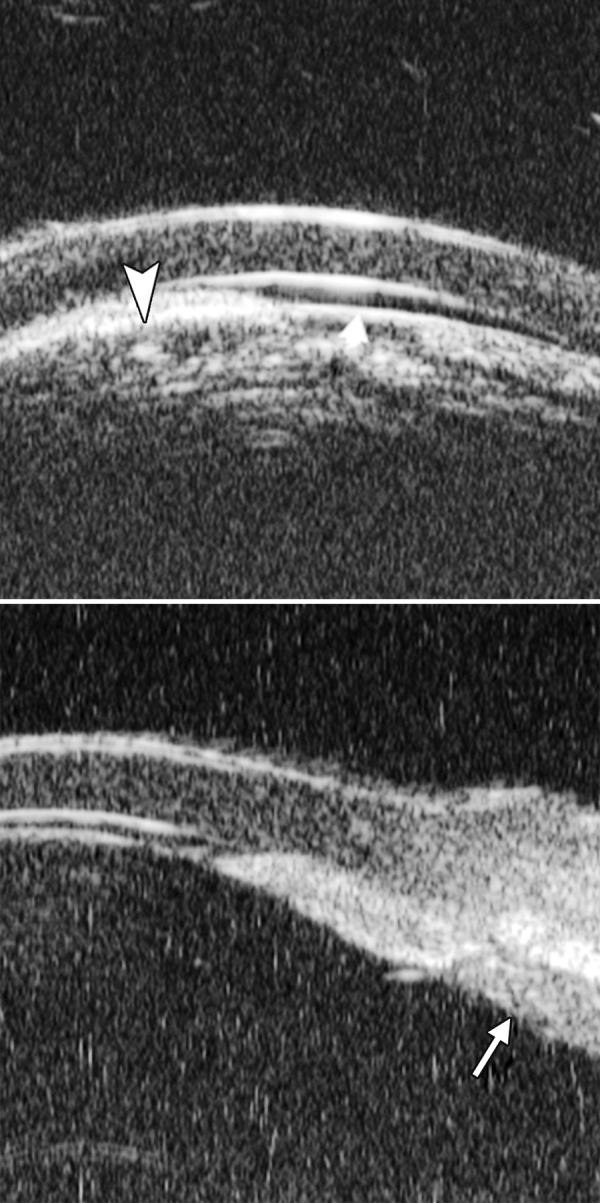
Malignant glaucoma

*Case summary:* A 52-year-old male presented at 2nd day after trabeculectomy. Slit lamp examination found central anterior chamber was of slit width with the anterior lens surface very close to corneal endothelium, IOP was 39 mm Hg. Bleb was diffused and slightly elevated. In Figure 27A, UBM examination revealed that the peripheral iris was closely contacted with the back of cornea (white arrowhead), anterior lens surface was very close to corneal endothelium (white arrow). In Figure 27B, ciliary body is compressed to become thin and long (white arrow).

7.    Congenital glaucoma

*Case summary:* A 2-year-old boy was diagnosed with bilateral congenital glaucoma. UBM revealed the typical spike-shape of the scleral spur disappeared (marked by black arrow), iris is thin and loss of stroma elasticity, peripheral iris was adhered on the back of the cornea. Other developmental abnormalities of trabecular meshwork could also affect the aqueous outflow. UBM is useful in detecting the changes of drainage angles particularly in those with cloudy cornea, but a general anesthesia is required for young patients affect its usage in congenital glaucoma.

**Fig. 28:**
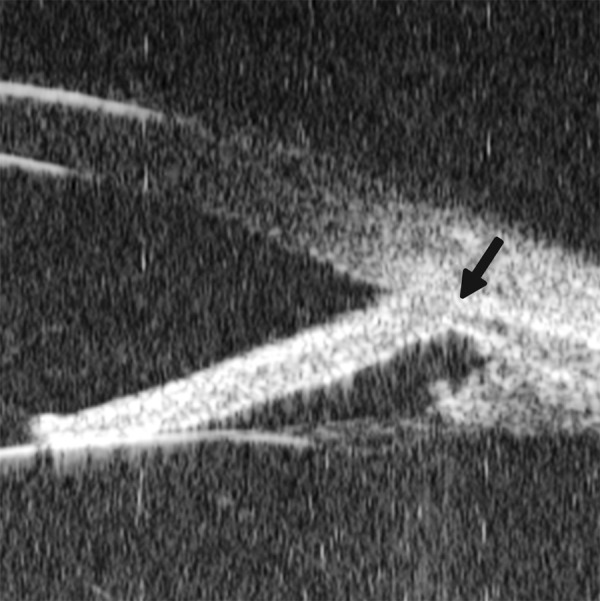
Congenital glaucoma

**Figs 29A to C:**
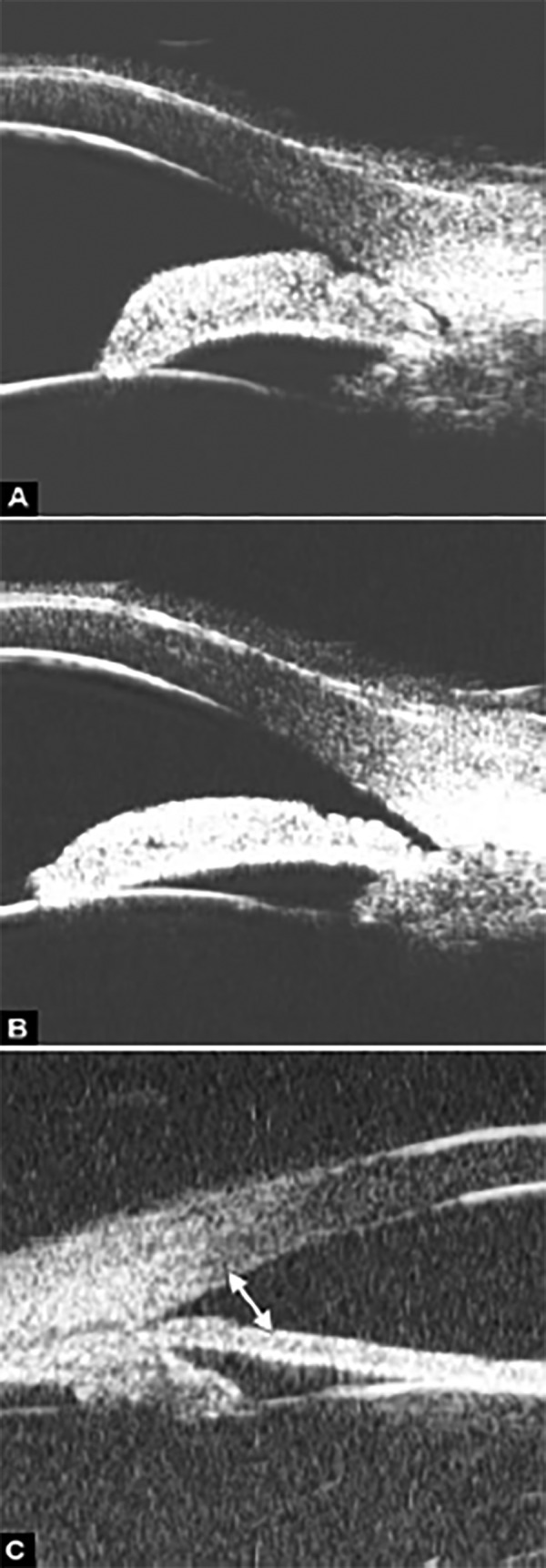
Laser iridotomy: Changes of drainage angle before and after treatment. (A) The iris appears bombe leading a narrow angle (white arrow) before LPI. (B and C) With a patten PI, the chamber angle is widened obviously (white arrow), the bombe iris becomes flat

**Figs 30A to C:**
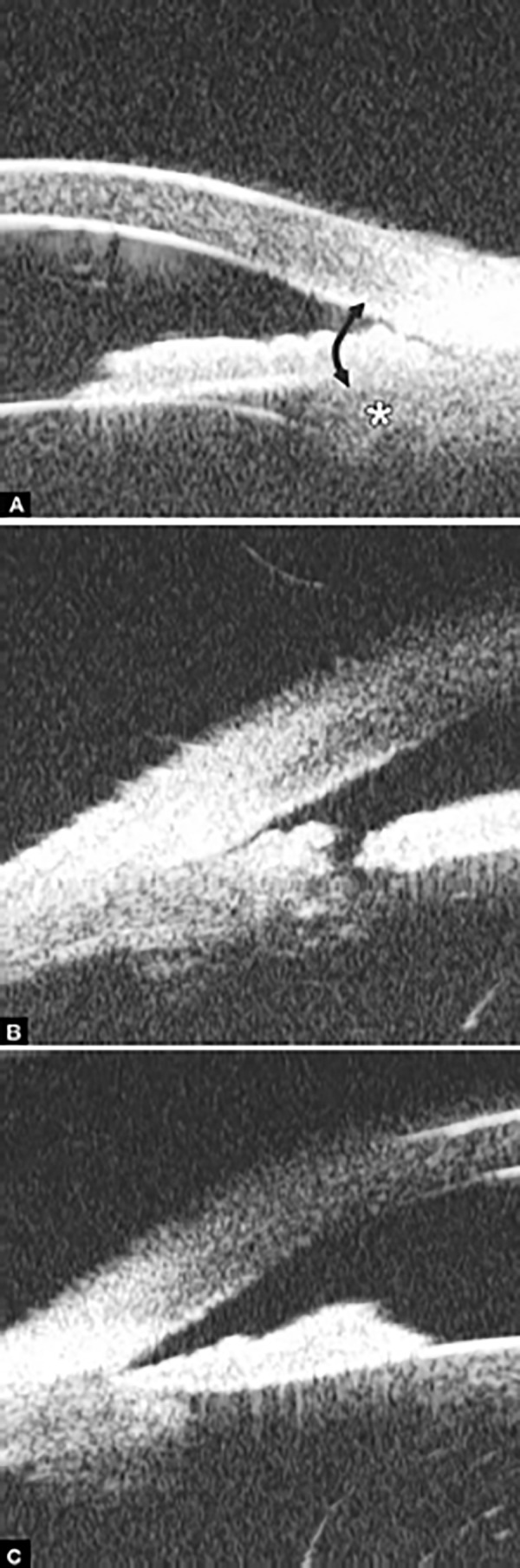
Iridotomy and iridoplasty in angle dominated by non-pupil block mechanism

## UBM IN GLAUCOMA SURGERY

### Peripheral Iridotomy and Iridoplasty

Peripheral iridotomy remains the cornerstone of the management of angle closure. It results in a significant increase in the angle width by eradicating the pupil block mechanism.

However, for those predominately caused by non-pupil block mechanism, peripheral iridotomy is not always effective. The following case was presented as anterior rotation of ciliary body (asterisk in Fig. 30A), drainage angle was not opened after laser PI treatment (Figs 30B and C). After that, the patient received iridoplasty and the angle was then opened (Fig. 30C).

### Cyclophotocoagulation

Laser cyclophotocoagulation is considered for as a safe cyclodestructive procedure for refractory glaucoma patients who have failed trabeculectomy or tube shunt procedure, or patients with minimal useful vision and uncontrolled IOP, or those being reluctant for surgery. Transcleral and endoscopic cyclophoto-coagulation are two procedures commonly used. Accurate positioning of the laser probe is challenging in the destructive eyes and complications, e.g. hemorrhage and severe inflammation are common. Endoscopic cyclophotocoagulation is an invasive procedure but allows visualization of the target tissues. UBM is useful in the observation of postoperative changes and identification of postoperative complications.

**Figs 31A and B:**
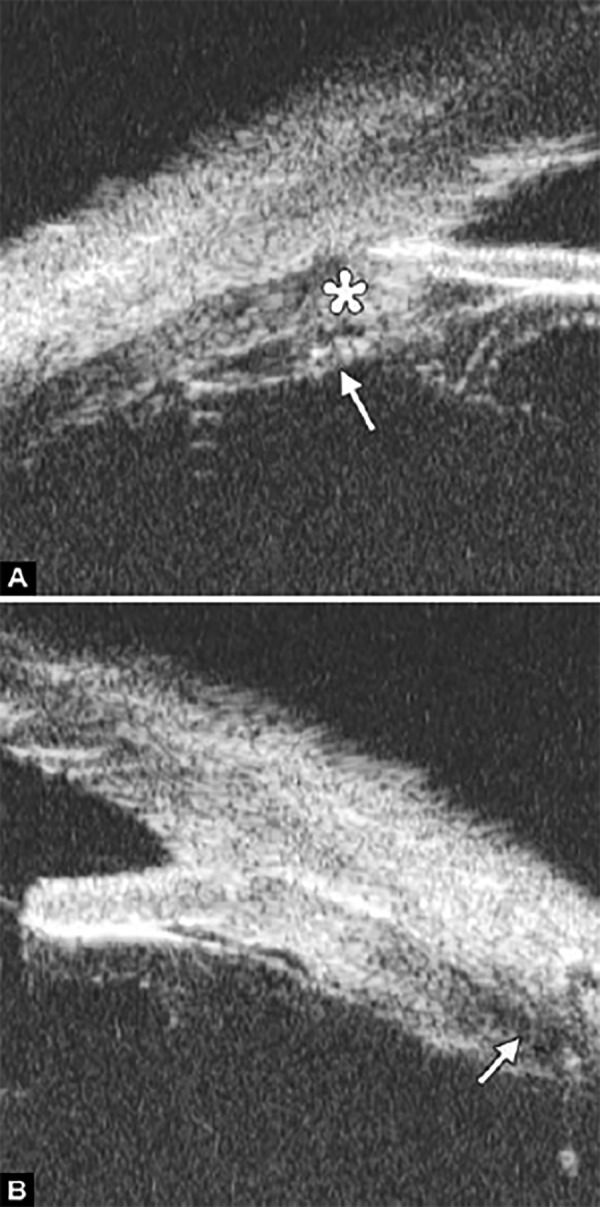
Cyclophotocoagulation

**Fig. 32:**
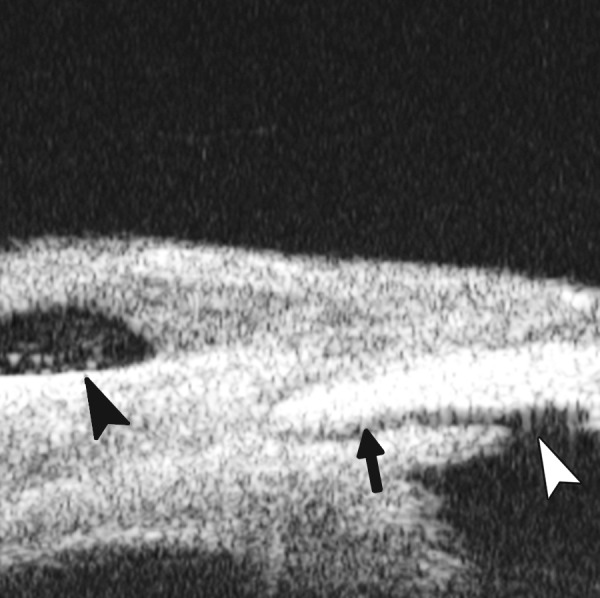
Normal filtering bleb (black arrowhead), unobstructed filtration channel (black arrow) connected with the inner filtering opening (white arrowhead)

**Fig. 33:**
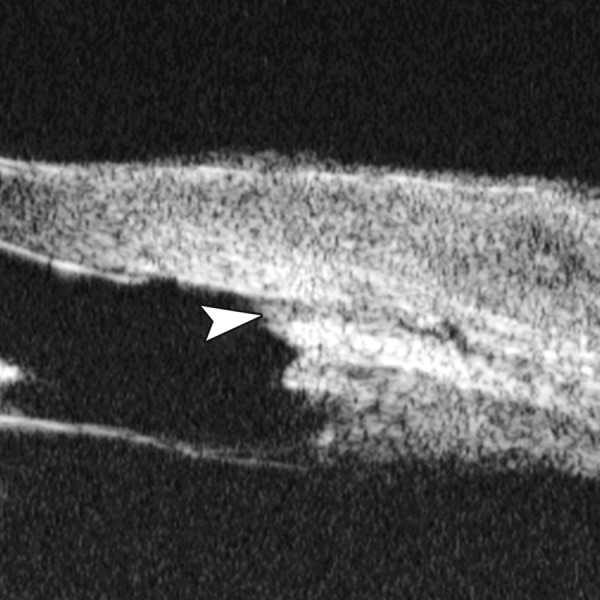
Obstruction of inner filtering opening (white arrowhead) which is usually led by the blood clot

**Fig. 34:**
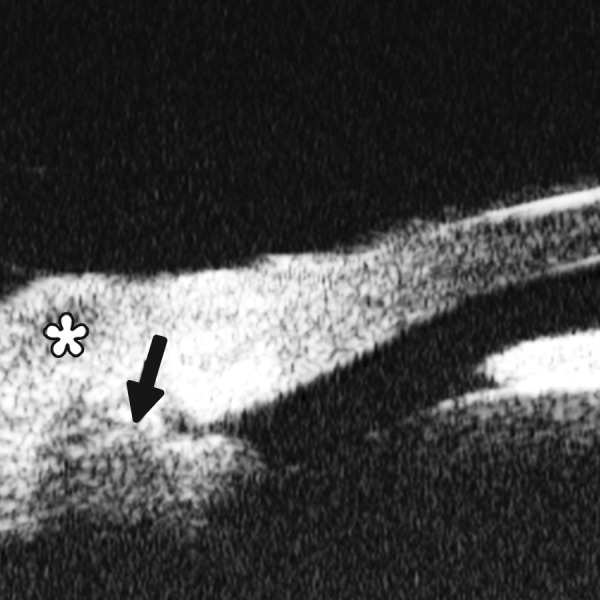
The inner opening is presented but no intrascleral pathway (black arrow) and no fluid spaces in the bleb (asterisk)

**Fig. 35:**
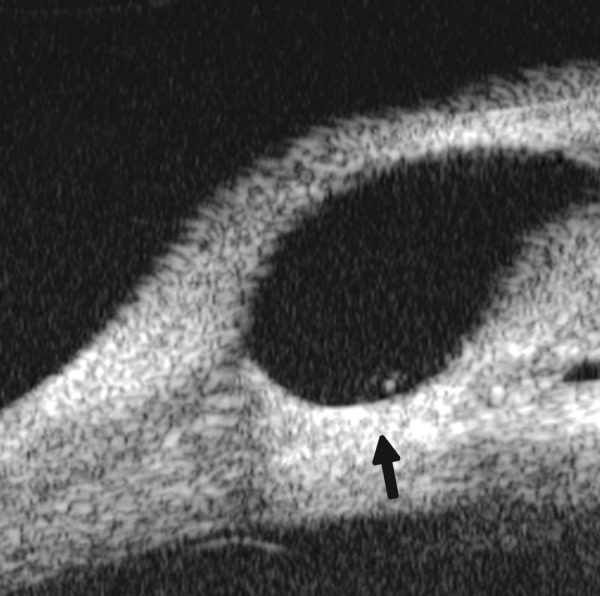
Scarring is presented at the bottom of the bleb (black arrow) that obstructs the effective outflow to conjunctiva

**Fig. 36:**
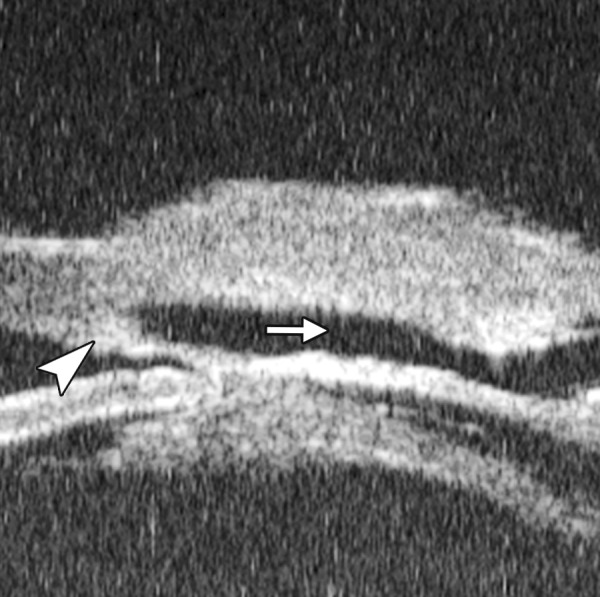
An intact thin trabeculo-descemetic membrane (white arrowhead), an intrascleral lake and pathway under the scleral flap to subconjunctiva cavity (white arrow) in nonpenetrating trabecular surgery

**Fig. 37:**
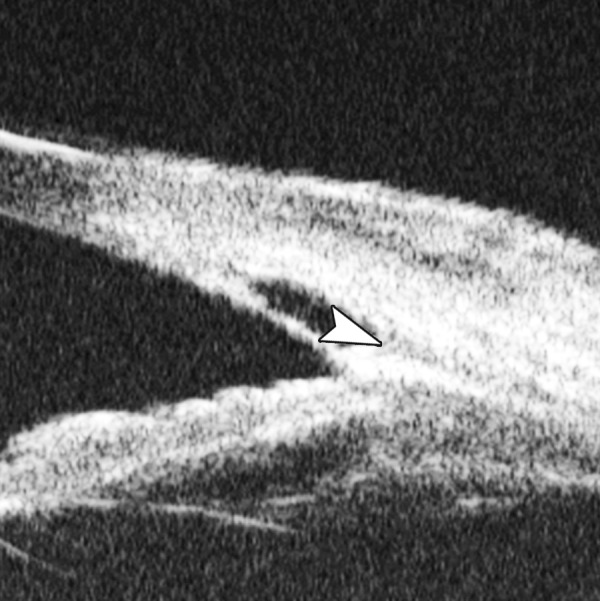
Exudation in the intrascleral cavity (white arrowhead) is usually formed by blood clot, which will decrease the aqueous outflow

**Fig. 38:**
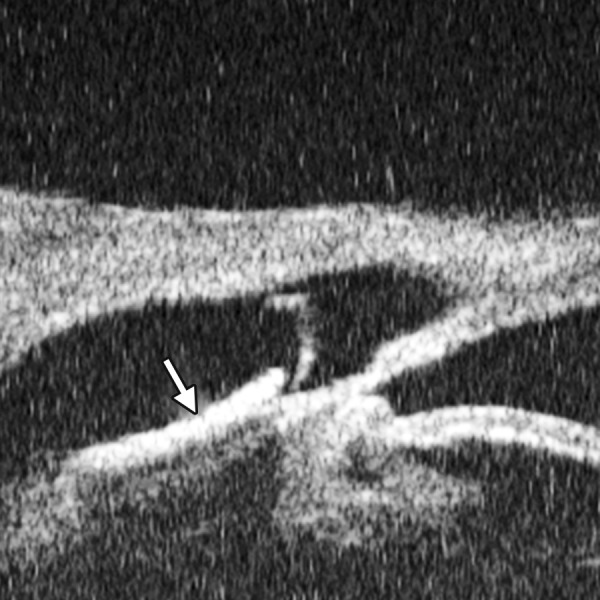
Viscoelastic material in the intrascleral cavity (white arrow) which is used to prevent scleral flap adherence and maintain the filtration cavity

**Figs 39A and B:**
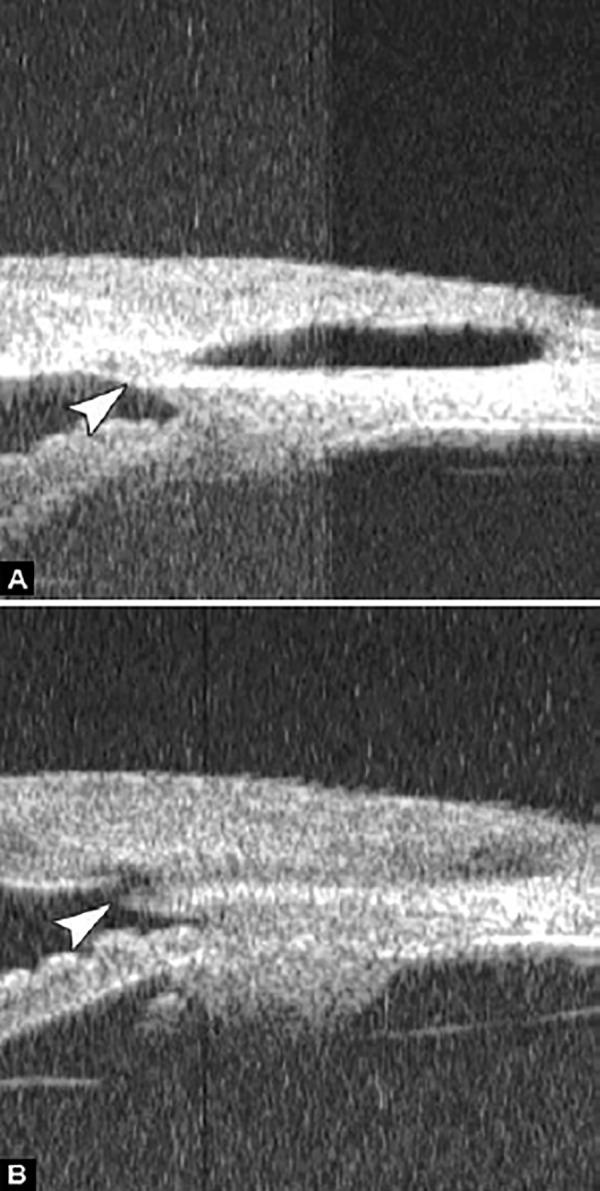
(A) The thick trabeculo-Descemetic membrane (white arrowhead) obstructed the outflow and compromise the IOP control. (B) After the membrane was broken by laser goniopuncture, the aqueous can be drained through the gap (white arrowhead), and the IOP was controlled in normal range

Case Summary

A 54-year-old man presented with visual acuity finger counting, IOP 45 mm Hg. Primary diagnosis was secondary glaucoma due to emulsified silicone oil in the anterior chamber. One week after the transcleral cyclophotocoagulation, IOP was 30 mm Hg. UBM reveals the atrophy of ciliary body (asterisk in Fig. 31A), fibrovascular membrane on the surface of ciliary body (white arrow in Fig. 31A) and choroidal effusion were also presented (white arrow in Fig. 31B). This suggested strong inflammation response of the treatment.

### Trabeculectomy

Trabeculectomy is one of the most widely performed glaucoma surgery procedures for glaucoma. UBM allows visualizing the status of surgical openings, outflow channel and filtering bleb. Thus, it is useful for identifying the causes of operation failure.

### Nonpenetrating Trabecular Surgery

Recently, there has been renewed interest in nonpenetrating trabecular surgery because of the advantage to avoid potential complications related to ocular entry, such as early hypotony and cataract progression. A deep sclerectomy is used to dissect the scleral tissue and unroof the Schlemm’s canal and Descemet’s membrane is separated from corneoscleral junction, resulting a thin but intact window to anterior chamber. Non-penetrating trabecular surgery is not as successful as trabeculectomy in reducing IOP. UBM is useful as being able to visualize the floor of sclerectomy, the intrascleral cavity and subconjunctival filtration (not visible during slit lamp examination) and thus able to assess the factors leading to surgical failure.

**Figs 40A and B:**
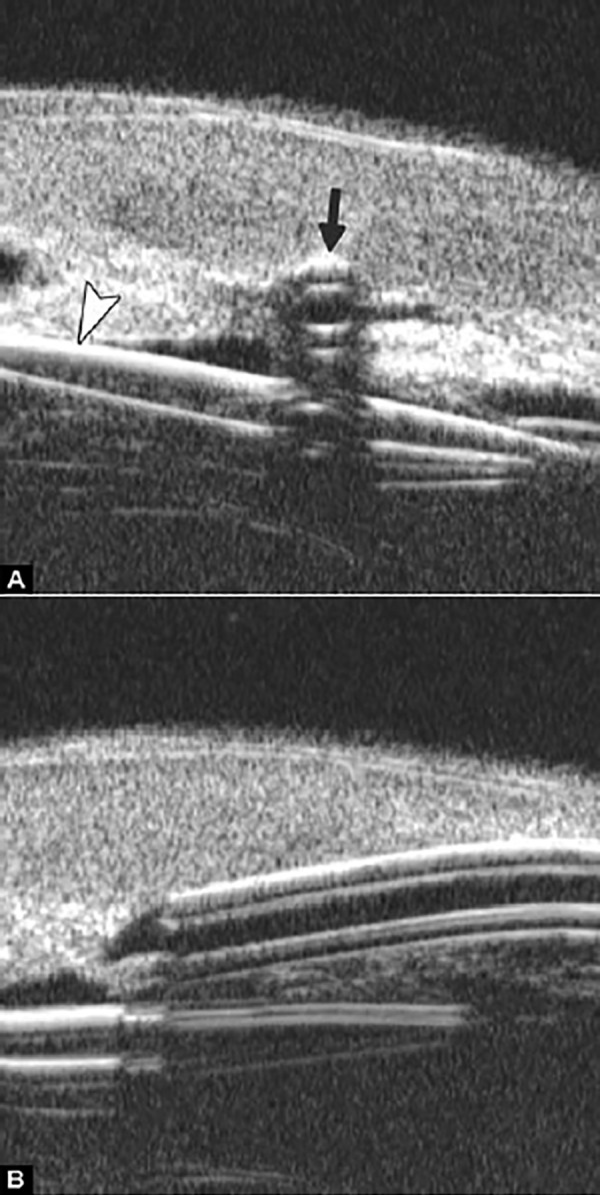
Shunt implant

### Shunt Implant

Case Summary

A 56-year-old man was diagnosed as neovascular glaucoma. Phaco and IOL were performed previously on this eye. Two days after shunt implant surgery, hyphema occurred and obscured the visibility of drainage device. UBM revealed a cross-sectional (black arrow in Fig. 40A) and sagittal section view (black arrow- head in Fig. 40A) of the implant. Opening of the implant was obstructed by blood clot, which suggested the neovessels’ bleeding affect the normal outflow.

### Lens

1. Morphological abnormalities of the lens
2. Lens subluxation
3. Lens-related surgery.

Normal Intraocular Lens

Complication of Intraocular Lens Implantation (Fig. 43A)

Iris Changes after IOL Implantation

(Iris Bombe or Perforation)

*Case summary:* A 65-year-old male presented as blurring in the left eye which has had phaco combined with IOL implantation surgery 4 years ago. Slit lamp revealed slight corneal edema, iris root dialysis at 11 o’clock with IOL loop exposed. UBM demonstrated the highly reflective image of IOL loop incarcerated at iris root (white arrow). Fibrous exudation was also presenting in the corresponding angle (asterisk).

**Fig. 41:**
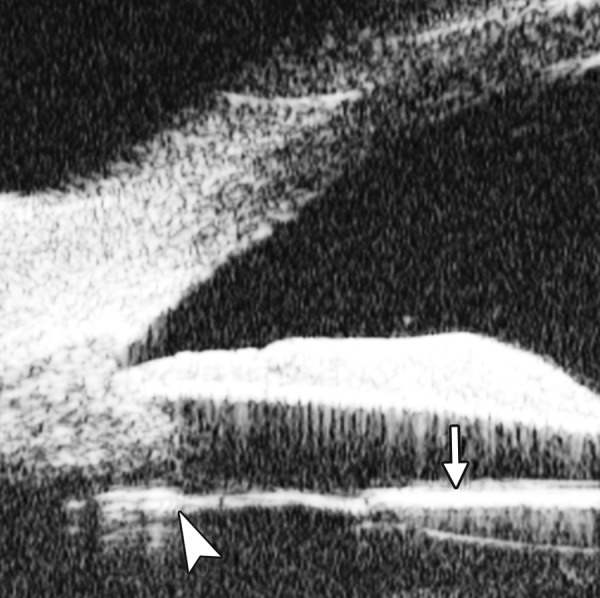
Cross-sectional image of posterior chamber. Intraocular lens includes body (white arrow) and loop (white arrowhead)

**Figs 42A and B:**
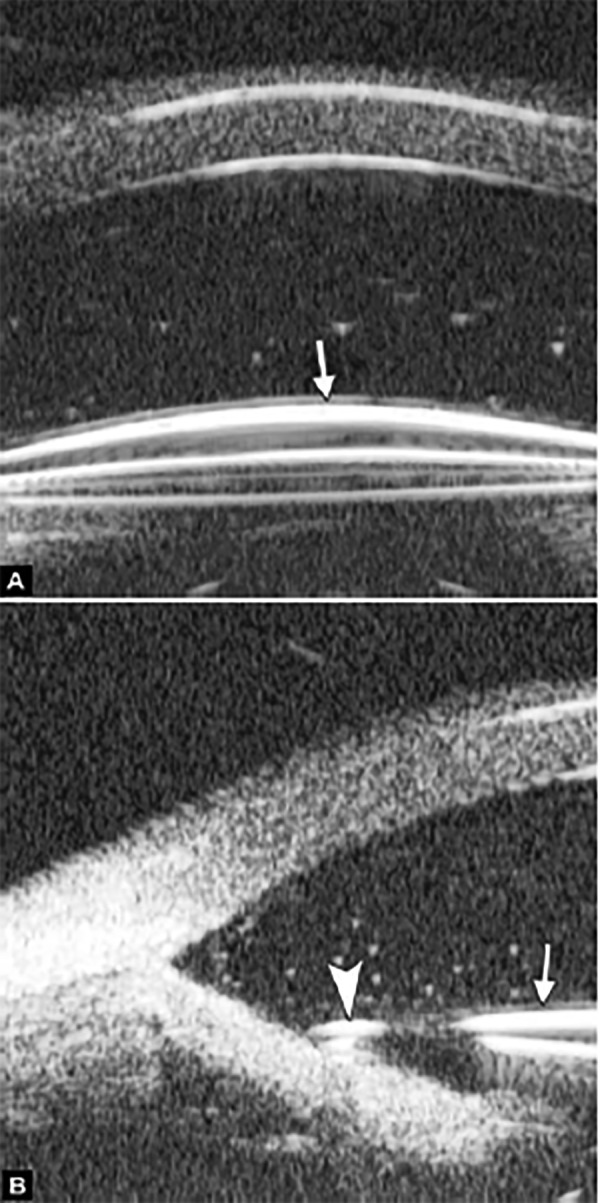
Cross-sectional image of anterior chamber. Intraocular lens includes body (white arrow) and loop (white arrowhead)

Residual Lens Cortex after Cataract

Capsular Block Syndrome

Capsular block syndrome (CBS) is a complication associated with anterior continuous curvilinear capsulorhexis (CCC). When the size of CCC is excessively small then comparing to IOL optic diameter, the IOL optic occlude the CCC opening which may result in a blockage of the content within the capsular bag. Relatively high osmotic materials, such as residual viscoelastics can absorb aqueous through the lens capsule and make the capsular bag distended to an abnormal degree. The anterior displacement of the optic and iris diaphragm shallows the anterior chamber and may result in pupil block glaucoma. Patients with this syndrome may often have an unexpected myopic overrefraction and increasing the risk of posterior capsular opacity.

**Figs 43A and B:**
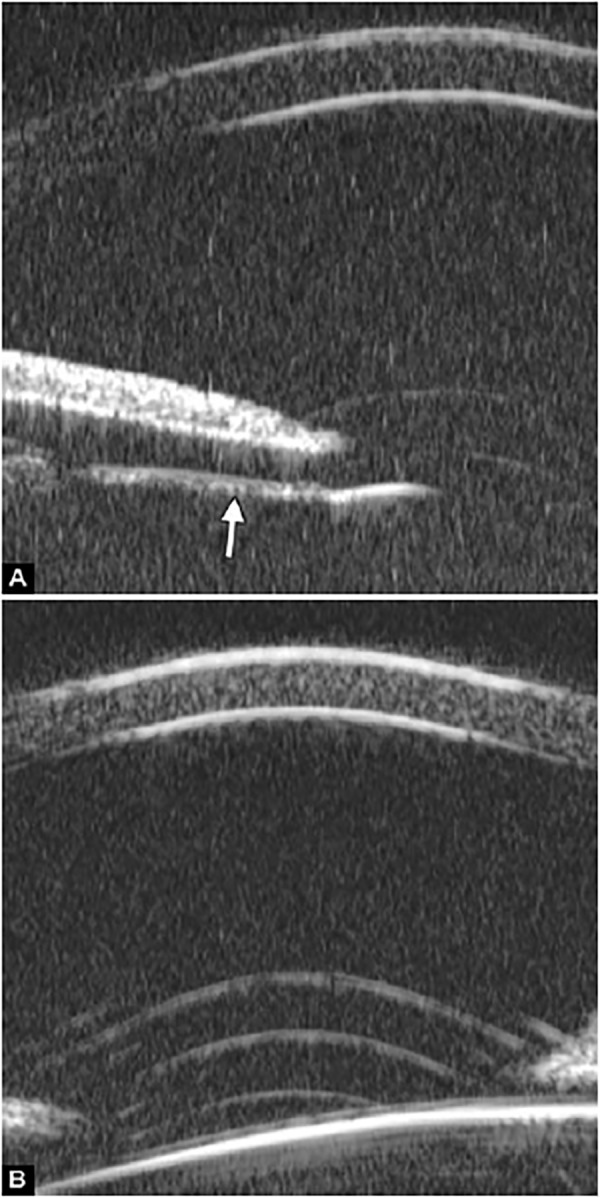
Intraocular lens dislocation: (A) the IOL dislocated to underneath the iris (white arrow) with the loop exposed in pupil area, (B) the IOL dislocation in sagittal axial, presenting as IOL lying at different distance to iris on two polars

**Fig. 44:**
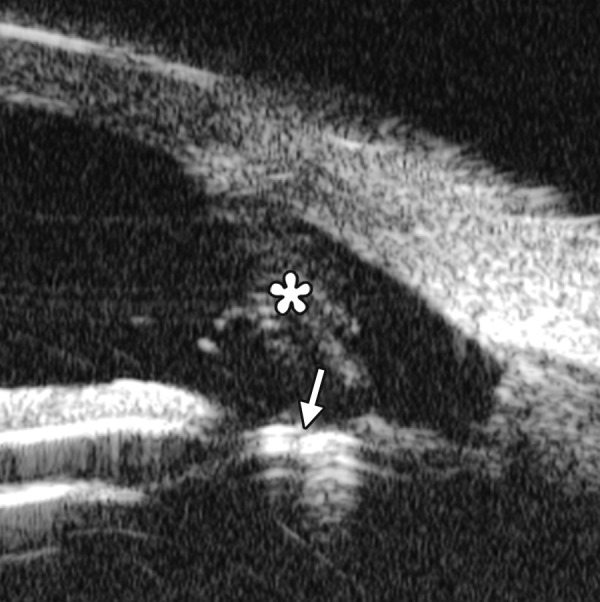
Complication of iris perforation by IOL loop

**Fig. 45:**
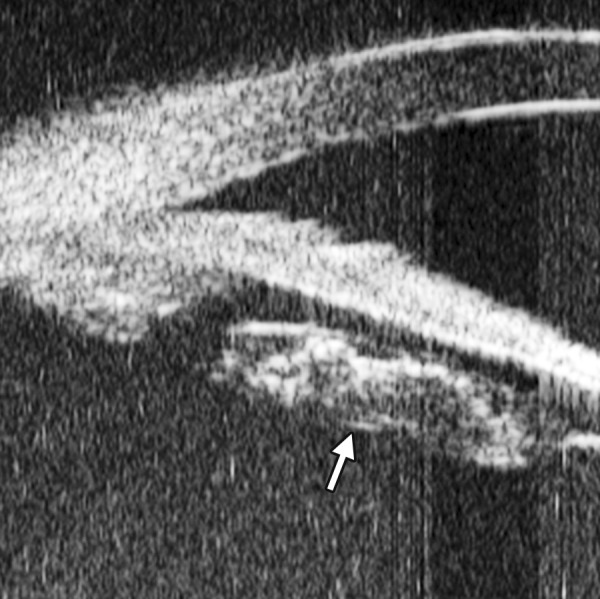
Residual lens cortex after IOL implantation, presenting as distinctly outlined mass with irregular reflectivity located at equator position of lens (white arrow)

**Figs 46A and B:**
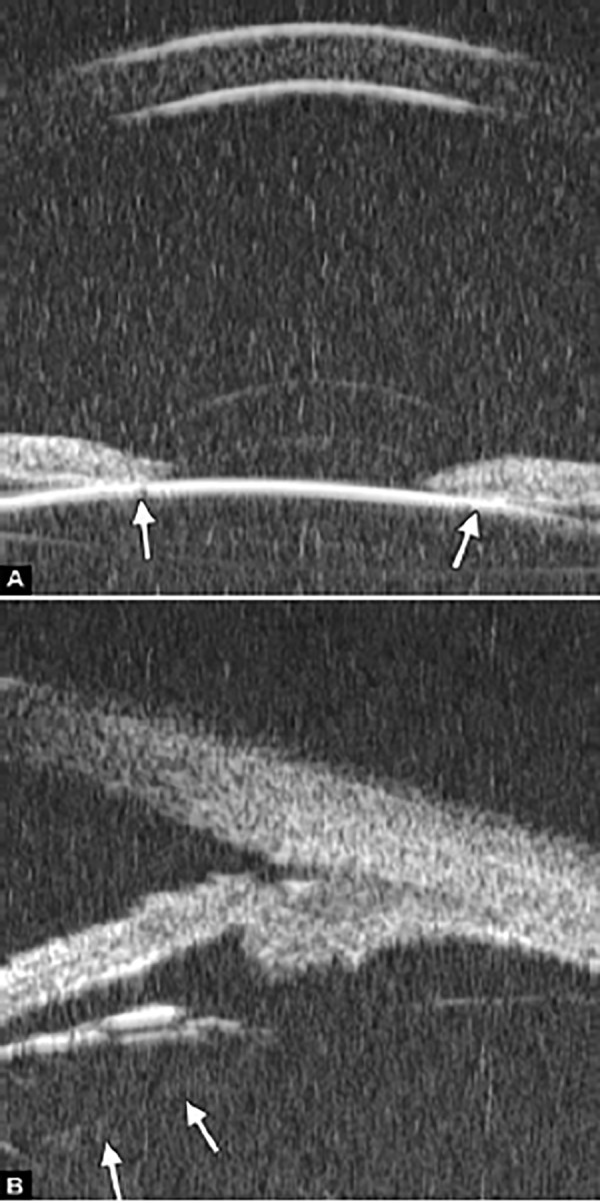
Capsular block syndrome

**Figs 47A and B:**
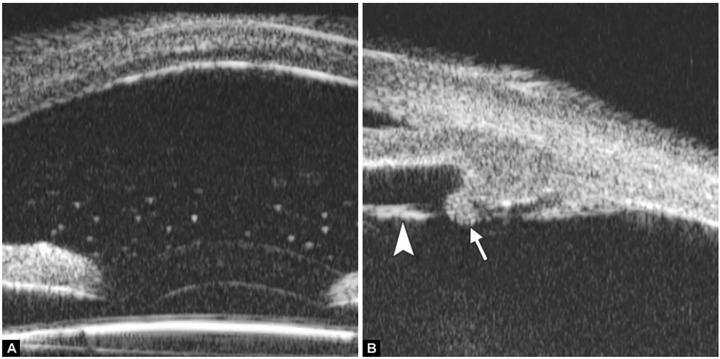
Anterior uveitis

**Figs 48A to D:**
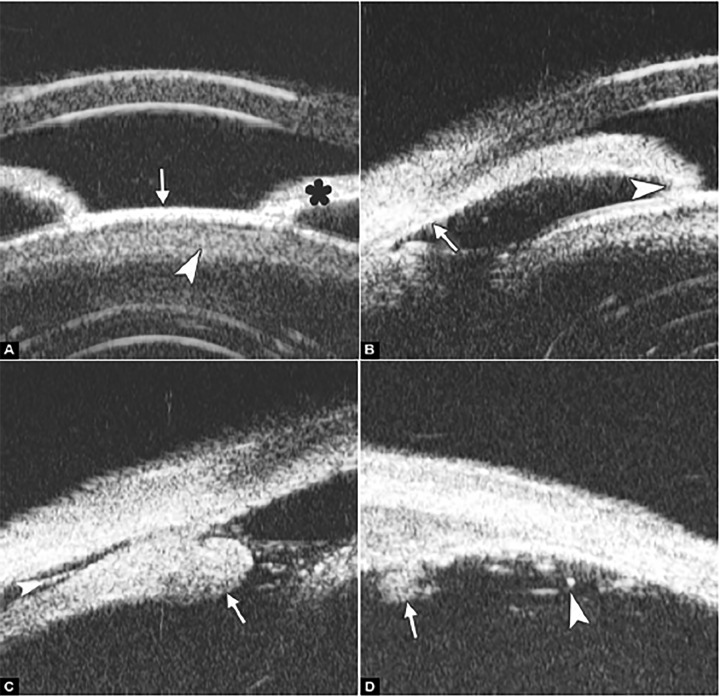
Intermediate uveitis

*Case summary:* A 67-year-old man presented 6 months after phaco/IOL surgery with progressive visual blurring. Visual acuity was 0.3, IOP was 18 mm Hg. Slit lamp examination found that the diameter of CCC opening was approximately 4 mm, which was completely occluded by IOL optic. UBM revealed the close contact between IOL optic and anterior capsule (white arrow in Fig. 46A), backward distended posterior capsule was also observed (outlined by white arrow in Fig. 46B).

### Uvea

Anterior Uveitis

*Case summary:* A 46-year-old female presented as having pain, redness, photophobia and blurring for 3 days. Primary diagnosis was anterior uveitis. Slit lamp examination found conjunctival injection, corneal edema, keratic precipitates, anterior chamber flares UBM demonstrated high reflectivity floaters in the anterior chamber (Fig. 47A), slight edema of ciliary body covered by exudative material (white arrow in Fig. 47B) as well as the inflammatory exudation attached on the zonules (white arrowhead in Fig. 47B).

Intermediate Uveitis

Intermediate uveitis is an insidious type of uveitis characterized by inflammatory cells in the anterior vitreous, inferior pars plana. Because it can be seen by routine examination, the patients are often presented as secondary cataract, iris synechiae, vitreous floaters and cystoid macular edema. UBM is able to detect these changes and therefore able to provide further evidence for the diagnosis.

*Case summary:* A 46-year-old female presented as having recurrent redness and blurring. Visual acuity was hand motion and IOP was 35 mm Hg. Slit lamp examination demonstrated shallowing of anterior chamber, pupil membrane, iris convex and significant lens opacity.

UBM imaging (Fig. 48A) evidenced shallowing of anterior chamber and iris convex (black asterisk), pupil area is covered by a piece of exudative membrane (white arrow) and secondary lens opacity at anterior cortex (white arrowhead). Figure 48B demonstrated synechiae of anterior drainage angle (white arrow) and iris posterior synechiae (white arrowhead). Figure 48C provided that inferior ciliary body was edema and covered by exudative material and membrane (white arrow) and inflammatory exudation in suprachoroidal lumen (white arrow-head). In Figure 48D, inflammatory exudation is also found covering the pars plana (white arrow) and peripheral retina (white arrowhead).

Congenital Aniridia

*Case summary:* A 9-year-old boy presented with photophobia and blurring. Visual acuity was 0.01 and refraction was +4.50D correctable to 0.3. Slit lamp revealed bilateral nystagmus, iris was absent from 2 to 4 clock hours, residual underdeveloped iris was presented from 10 to 2 clock hours. UBM revealed similar result.

**Figs 49A and B:**
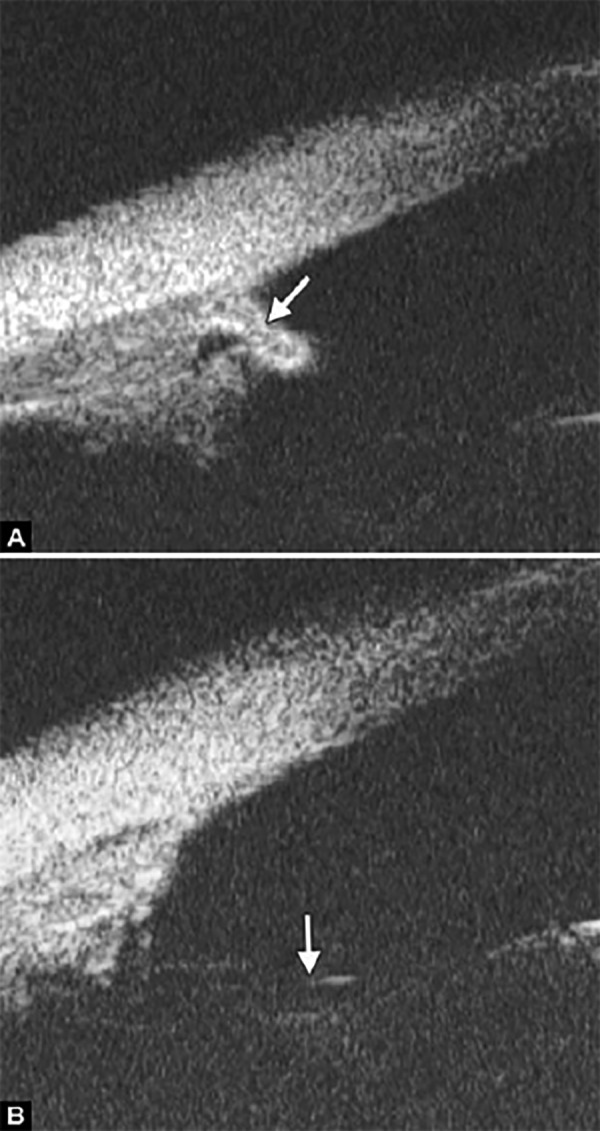
Aniridia: (A) revealed maldeveloped iris (white arrow) together with malformation of ciliary body, (B) revealed absolutely iris missing with zonules exposed (white arrow)

**Fig. 50:**
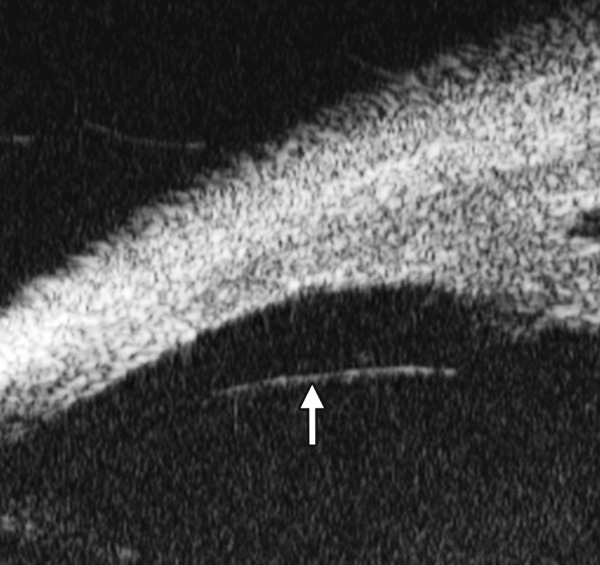
Normal vitreous and retina

**Figs 51A and B:**
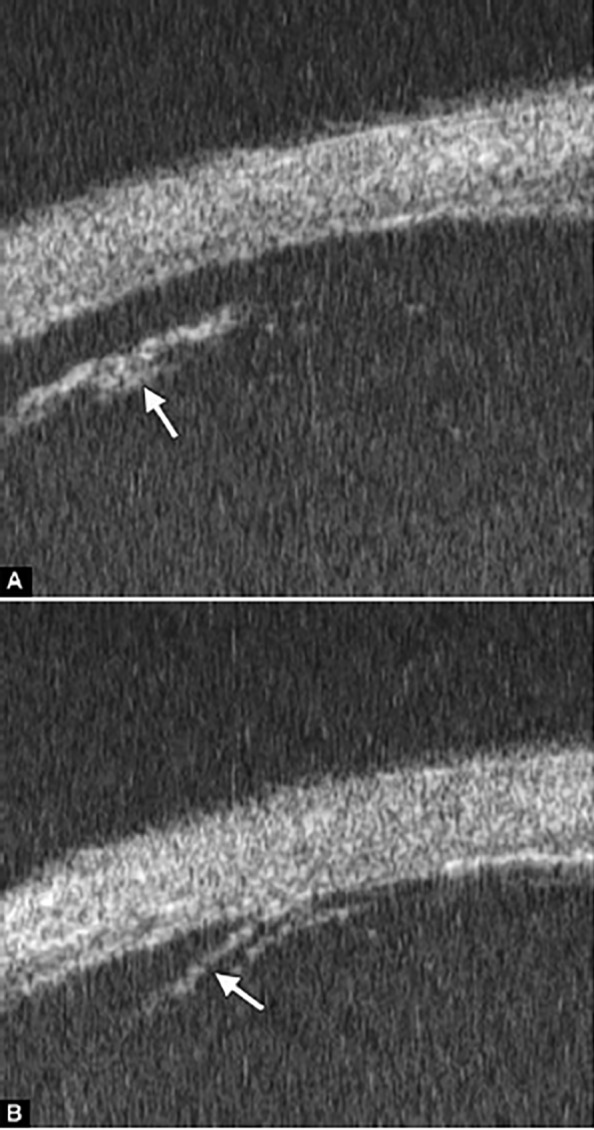
Pars plana dialysis

**Figs 52A and B:**
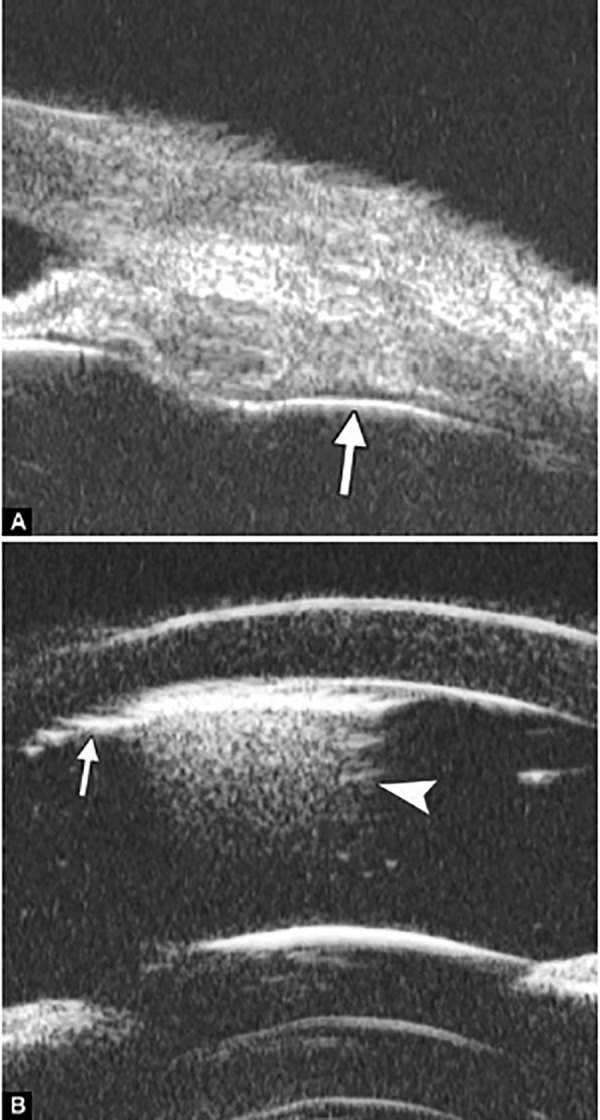
Silicone oil: (A) the highly reflective line (white arrow) is the interface of silicon oil, (B) Silicone oil emulsified in anterior chamber. The interface is high reflection (white arrow) with echoic shadow underneath it (white arrowhead)

### Vitreous and Peripheral Retina

Normal Vitreous and Peripheral Retina

Normal pars plana, ora serrata and peripheral retina appear consecutive and smooth in UBM image. The highly reflective line is the anterior border of vitreous (white arrow).

Retinal Detachment (Pars Plana Dialysis)

UBM can detect retinal detachment located in the anterior part of vitreous cavity. In Figure 51A, the line represents the free end of detached retina in pars plana dialysis (white arrow). The fine line (white arrow in Fig. 51B) reveals retinal detachment at very peripheral part of the fundus, which may not easily observed when the pupil is not dilated big enough.

Retina Surgery

Complications of Retinal Surgery

Anterior proliferative vitreoretinopathy (aPVR) is one of the common causes leading postoperative retinal redetachment. Construction of vitreous incarcerated at the pars plana punctures during the operation is thought to be the causation of the complication. Compared with three-mirror examination after pupil dilation, UBM supplies a convenient way to detect inner opening of punctures.

**Fig. 53:**
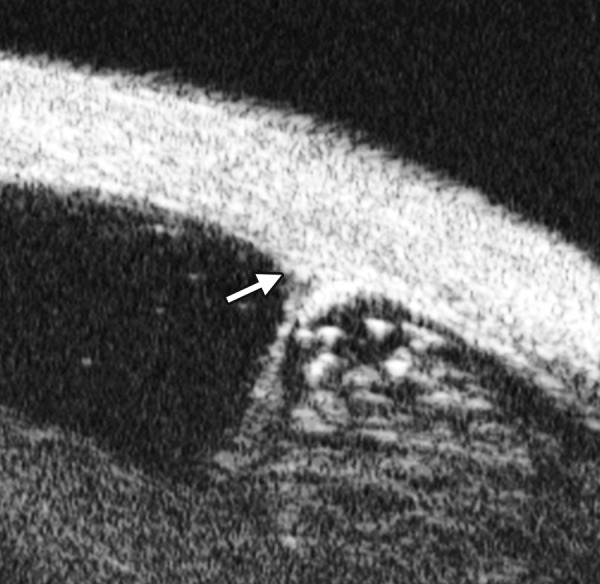
Anterior PVR. UBM images revealed fibrous strips incarcerated at the operation puncture (white arrow)

**Figs 54A and B:**
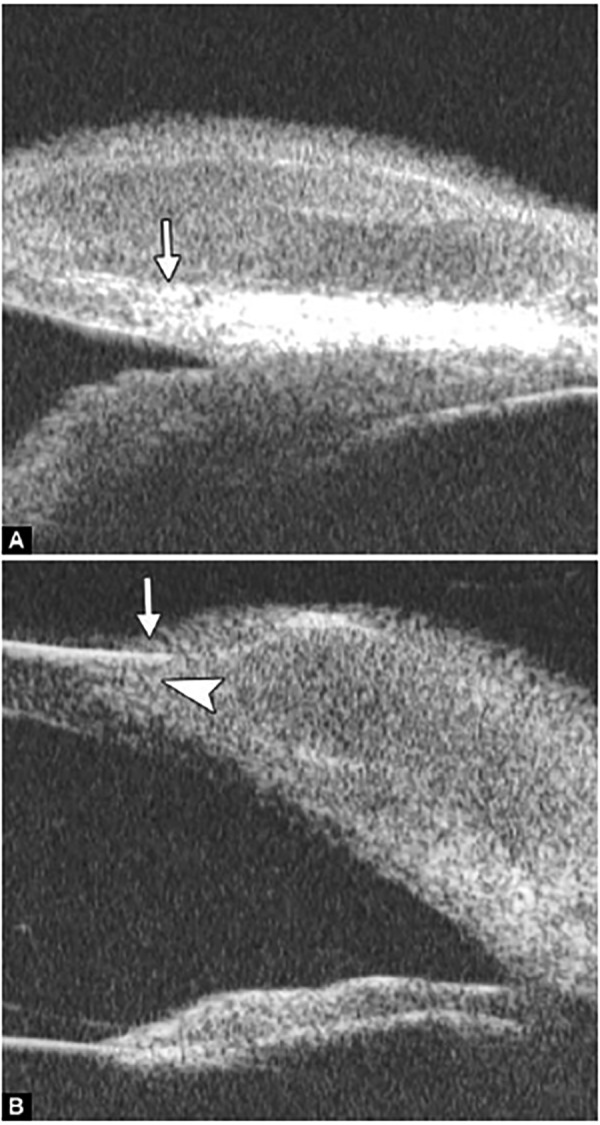
Limbal dermoid

### Anterior Segment Tumor

Conjunctiva Tumor

*Corneoconjunctival dermoid:* It is a congenital abnormality, neoplasm is mainly composed of fibrous and lipid tissue covered by epithelium. This neoplasm often affect conjunctiva or superficial layer of the cornea.

*Case summary:* A 9-year-old girl was diagnosed as limbal dermoid. Slit lamp found a round-shaped yellow nodule on the temporal limbus. UBM image (Fig. 54A) revealed a round neoplasm with uniform density. A clear boundary between the neoplasm and sclera is presented (white arrow) implying the tumor growing is self-limited without extension. Image (Fig. 54B) demonstrated the neoplasm has involved the superficial cornea (white arrowhead) leading local epithelium edema (white arrow).

**Fig. 55:**
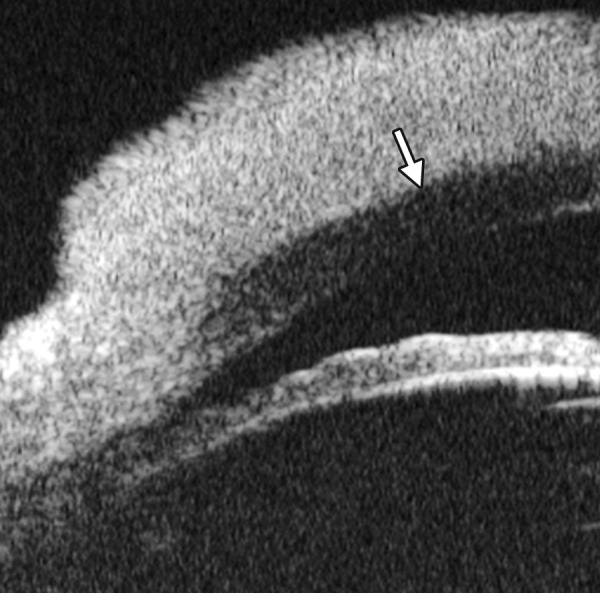
Corneal melanoma

**Figs 56A and B:**
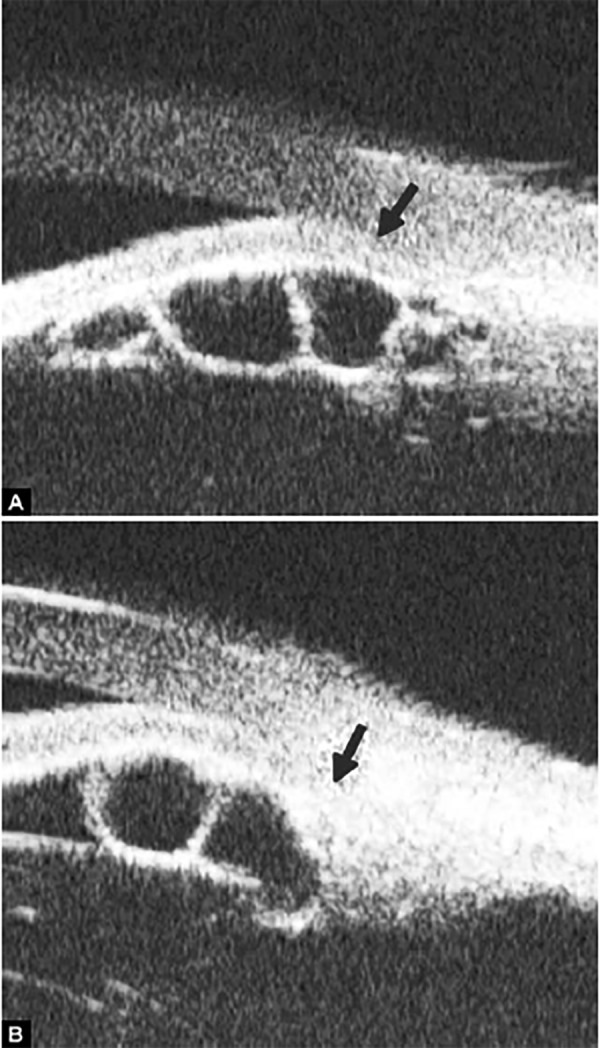
Iris cyst A and B show the right and left eye respectively

Corneal Neoplasm

*Case summary:* A 39-year-old man was diagnosed as corneal dermoid. Slit lamp examination identified a 4 × 4 mm demarcated dark neoplasm. UBM revealed a neoplasm of uniformly high reflectivity on the limbus, both the cornea and conjunctiva were involved but not yet invade Bowman’s membrane (white arrow). Pathological testing confirmed the diagnosis of melanoma. UBM is helpful to identify the extent of neoplasm and therefore help decide the surgical treatment plan.

**Fig. 57:**
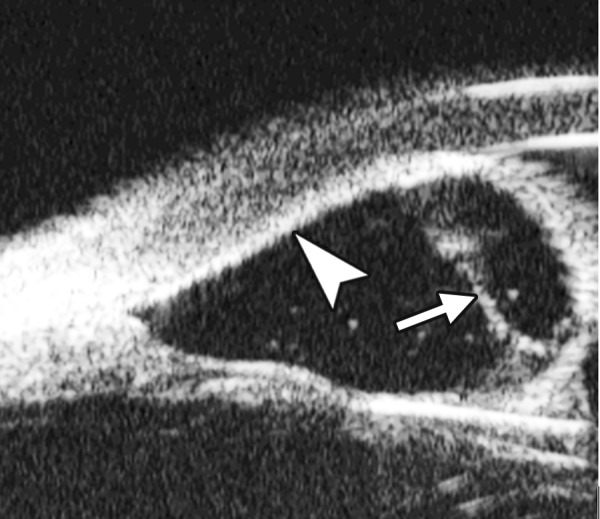
Iris implantation cyst. UBM revealed a large cyst on the superior quadrants with iris atrophy and localized iris-cornea contact (white arrowhead). The cyst had thin septum (white arrow) and scatter reflectivity inside. UBM is helpful to identify the extent of cyst as well as the relationship with neighboring tissues

**Fig. 58:**
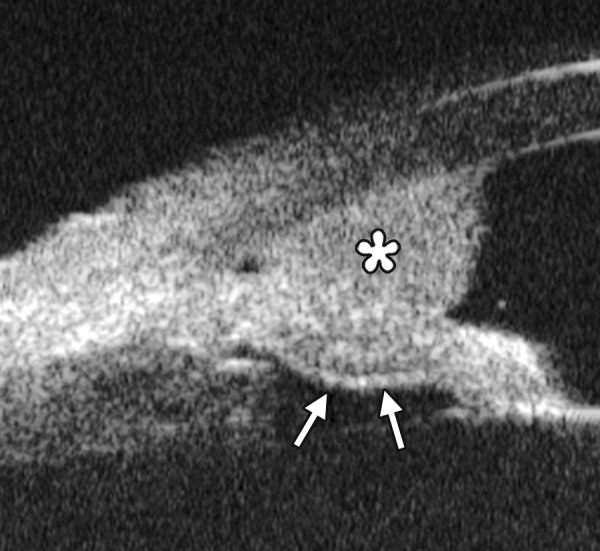
Iris melanoma

**Fig. 59:**
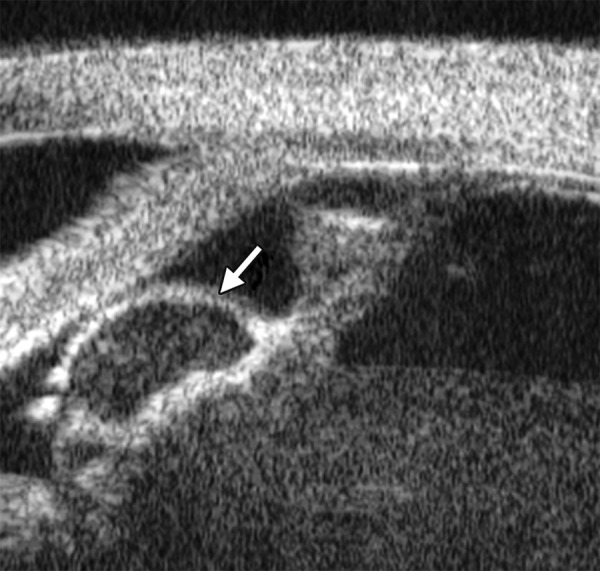
Ciliary body cyst

**Fig. 60:**
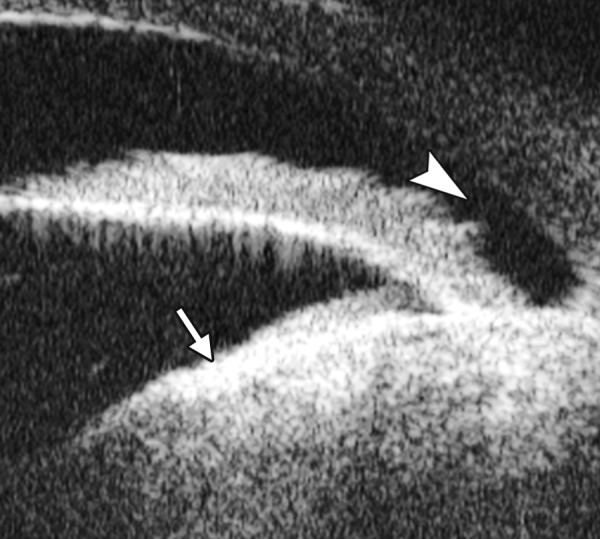
Ciliary body neurilemmoma. Neurilemmoma is one of the benign tumor of ciliary body. The UBM image shows a huge ciliary body mass with regular internal content (white arrow) which turns the iris closing to the cornea with angle entrance obviously narrowed (white arrowhead)

**Fig. 61:**
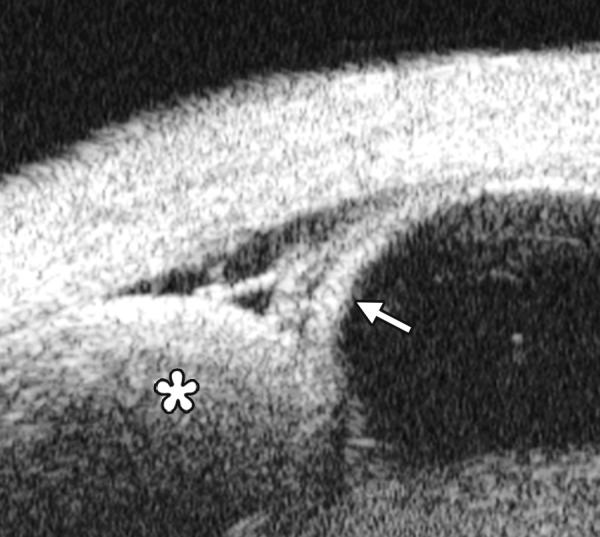
Choroidal melanoma. UBM examination shows the radial sectional image of a choroidal melanoma. A round shaped mass is noted extending centrally from posterior ocular wall (asterisk). Secondary exudative retinal detachment occurred at the peripheral fundus (white arrow)

Iris Neoplasm

Iris cyst (primary)

*Case summary:* A 60-year-old lady presented as having recurrent eye pain for 1 month. Visual acuity was 0.4 on right and 0.7 on left eye, IOP was 33 mm Hg on right and 18 on left eye. Slit lamp confirmed axial anterior chamber depth as 3 CT, peripheral chamber depth: Close on superior/inferior and nasal quadrants and 1/4 CT on temporal quadrant on right eye, peripheral chamber depth was 1/4 CT on all quadrants on left eye. UBM revealed multiple iris cysts on the back of the iris in all quadrants, which push the iris root upward causing secondary angle closure (black arrow).

**Figs 62A and B:**
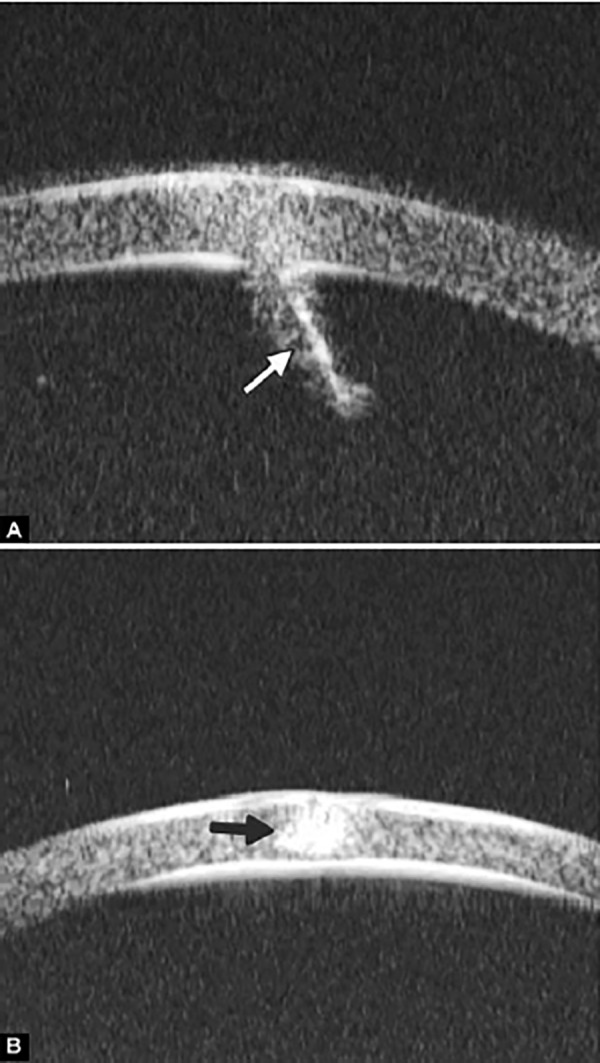
Cornea foreign body. (A) Fine iron wire (white arrow) and (B) tiny iron granula (black arrow)

**Fig. 63:**
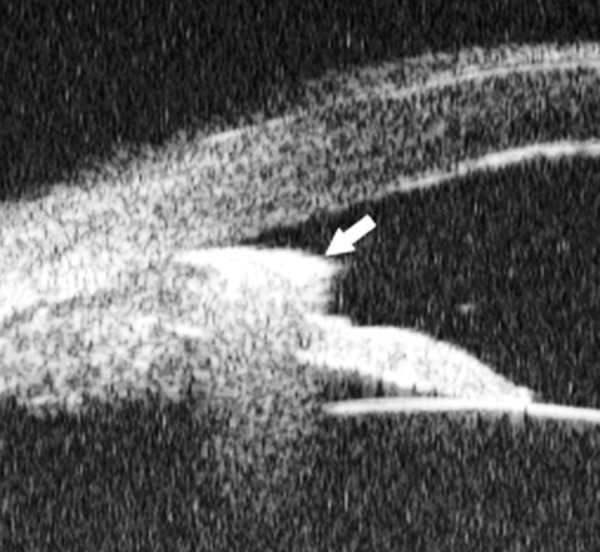
Anterior chamber angle foreign body. A piece of sharp metal debris was noted incarcerated in the chamber angle (white arrow). The shadow of the foreign body blurred the echo of iris beneath it

**Figs 64A and B:**
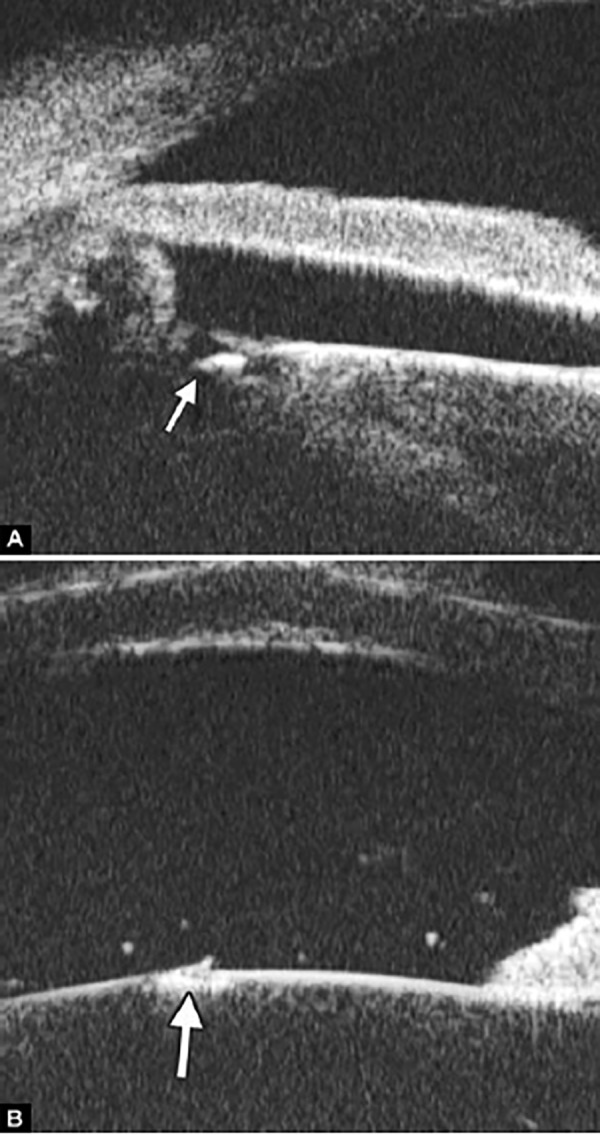
Lens foreign body. (A) A metallic hyperechoic foreign body was revealed lying at equater part of lens (white arrow), (B) the foreign body penetrated anterior capsule of lens and attached in the superficial cortex (white arrow)

Iris Implantation Cyst

*Case summary:* A 34-year-old female presented as having pain and blurring 3 years after extracapsular cataract extraction and IOL implantation. Visual acuity was 0.1 and IOP 40 mm Hg. Slit lamp examination confirmed corneal edema, peripheral chamber disappeared with iris synechiae at 9 to 2 clock hours.

Iris Melanoma

*Case summary:* A 70-year-old female presented with blurring in the right eye. Visual acuity was 0.2 and IOP 18 mm Hg. Slit lamp revealed a pigmented peripheral iris lesion in the superotemporal quadrant. The chamber angle was filled with the tumor from 1 to 3 O’clock. Tumor surface was attached on the corneal endothelium. UBM demonstrated, as shown in Figure 54, the tumor is of regular internal structure (asterisk). A fusiform thickening of the midperipheral portion of iris especially at the posterior stroma was noted under the tumor (outlined by the white arrows).

**Figs 65A and B:**
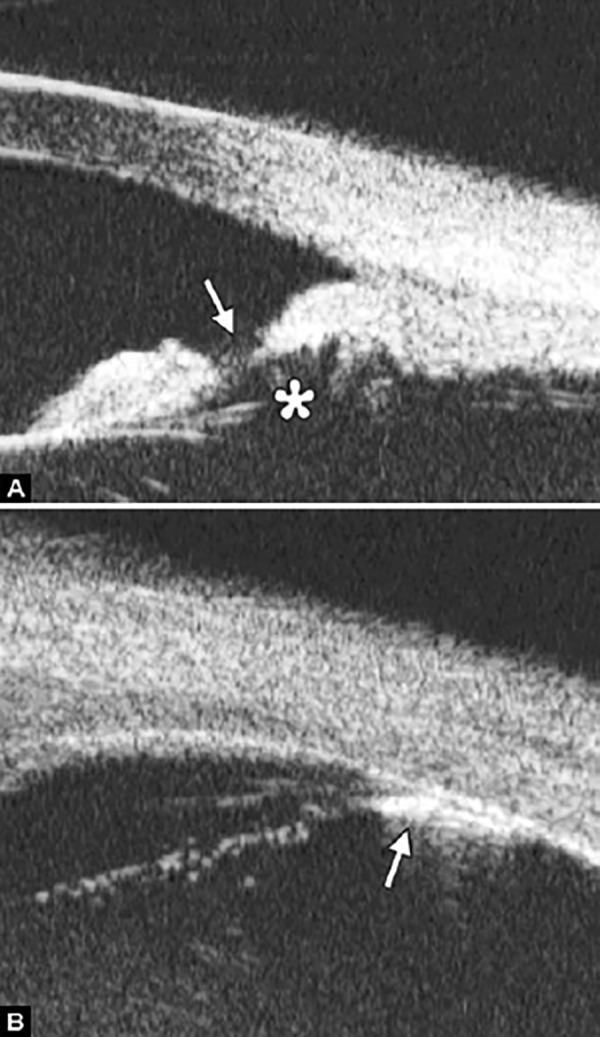
Anterior vitreous foreign body. (A) Reveals the pathway of foreign body, perforating iris (white arrow), passing through zonules (asterisk), and located in the vitreous at the ora serrata (white arrow in B)

**Fig. 66:**
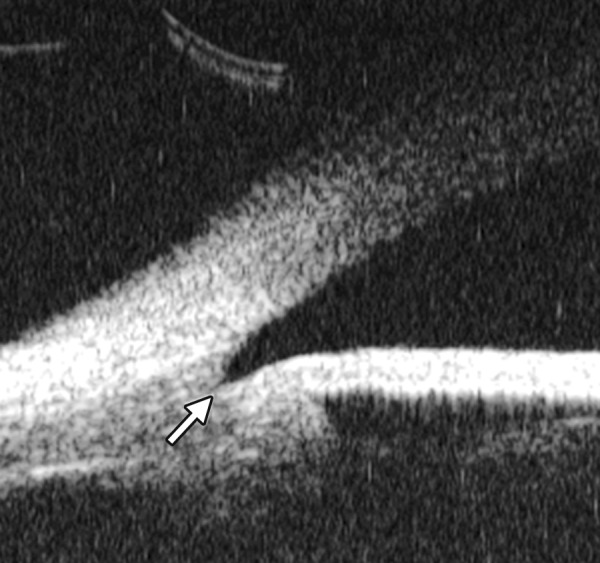
Angle recession usually occurs in eye contusion. The UBM hallmark of the lesion is laceration of angle apex (white arrow) with the ciliary body band abnormally widened

Ciliary Body Cyst

*Case summary:* A 46-year-old male presented with eye pain and blurring in the left eye. Visual acuity was 0.05 and IOP 30 mm Hg. Slit lamp examination revealed narrowing of chamber angle width from 7 to 12 O’clock meridian. UBM revealed a cyst of the ciliary body (white arrow) with hypoechoic regular content inside. The neoplasm pushed the iris anteriorly, causing local chamber angle narrowing, which led to IOP rising.

**Fig. 67:**
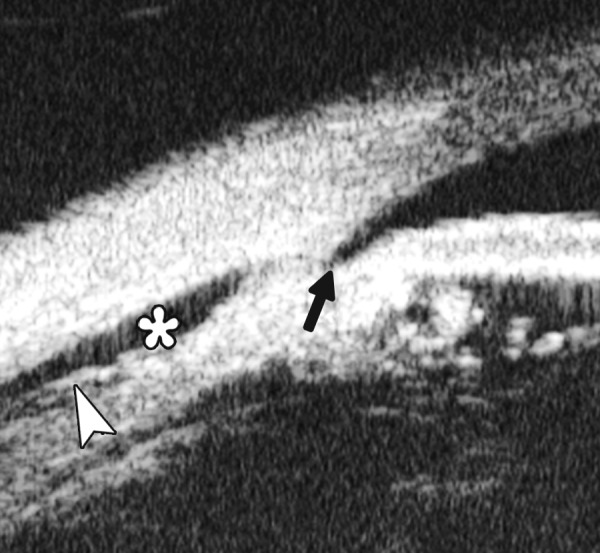
Ciliary body detachment in trauma is often led by suprachoroidal lumen hemorrhage or severe exudative inflammation of choroidal vessels. The UBM manifestation is dark space between choroids and sclera (asterisk) with highly reflective septum (white arrowhead) inside. Angle recession also occurred in this patient (black arrow)

**Fig. 68:**
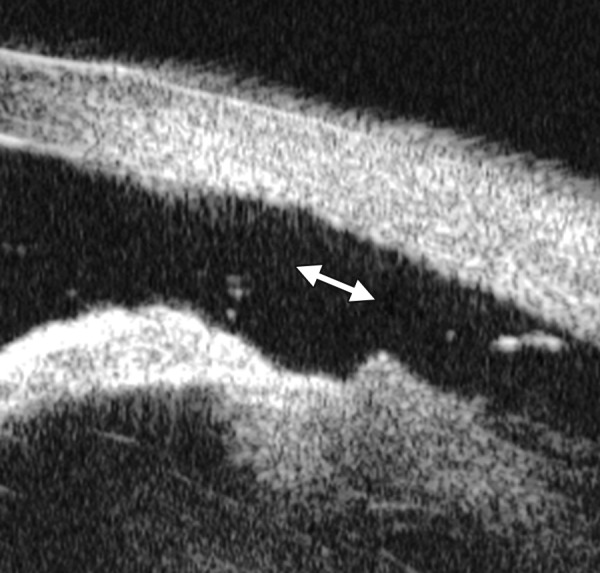
Ciliary body segregation is a severe form of ciliary body detachment when the external force is prompt and powerful. UBM hallmark of the lesion is a pathway connecting suprachoroidal lumen and anterior chamber (white arrows). The anterior part of uvea is totally separated from eyeball wall

### Eye Trauma

Intraocular Foreign Body

Lens luxation

**Figs 69A to C:**
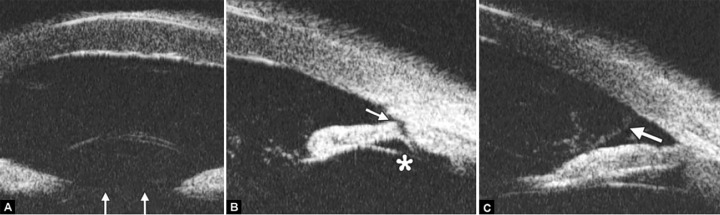
Lens subluxation

*Case summary:* A 29-year-old male was hit by fist 2 hours ago in the left eye, complaining of eye pain and blurring. Slit lamp examination revealed corneal edema, axial chamber was abnormally deepened. There was jelly like material incarcerated at the pupil edge with lens unobserved. In UBM images, the highly reflective line of lens surface is missing (white arrows in Fig. 69A), there is angle recession (white arrow in Fig. 69B) and the zonules disappeared (asterisk in Fig. 69B). In Figure 69C, vitreous mass can be observed in chamber angle (white arrow).

**Fig. 70:**
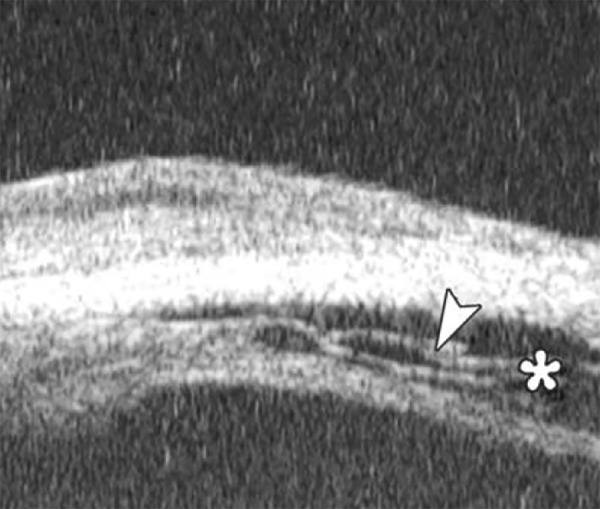
Choroidal detachment. The UBM manifestation is separated space (asterisk) between uvea and sclera with septum (white arrowhead), which is similar to that of ciliary body detachment except the location

### Anterior Segment Syndrome

Iridocorneal Endothelial Syndrome (ICE)

Iridocorneal endothelial syndrome (ICE) is a spectrum of diseases. There are three clinical subtypes: Chandler’s syndrome, Cogan-Reese syndrome and essential iris atrophy. Chandler’s syndrome is characterized by predominant corneal edema and mild changes of the iris. Cogan-Reese syndrome (iris-nevus) presents with the corneal findings and pigmented nodules on the iris. Essential iris atrophy presents with the corneal findings and corectopia (abnormal pupil position) with developments of stretch and melt holes with disease progression.

**Figs 71A and B:**
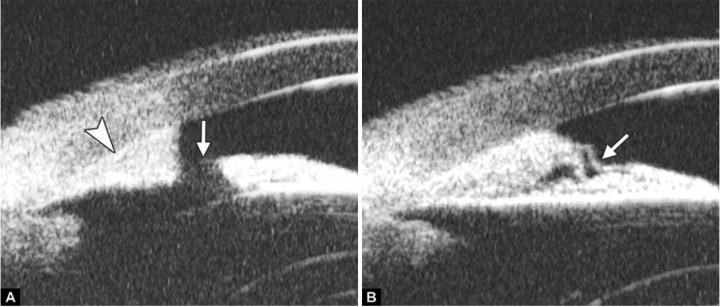
ICE syndrome. In UBM images, other than corneal edema, there is extensive PAS (white arrowhead in A), together with iris dystrophy, manifested as penetrating or laminal hole formation (white arrow in Figs A and B)

*Case summary 1:* A 38-year-old man presented with progressive visual loss and pain for 1 year. Visual acuity was 0.01, IOP 32 mm Hg. Slit lamp examination identified corneal edema with bullous keratopathy and normal anterior chamber depth. Extensive peripheral anterior synechiae and multiple iris holes were presented. Corneal endothelial examination did not identify endothelial cells with normal morphology. Primary diagnosis was ICE syndrome.

*Case summary 2:* A 23-year-old female presented with blurring in right eye for 6 months. Visual acuity was 0.05 with IOP 45 mm Hg. Slit lamp examination revealed edematous cornea, pupil dislocation. Iris was considerably thin with extensive peripheral synechiae. Fundus examination found CDR 0.8, pale optic disk and glaucomatous cupping. UBM examination confirmed the thinning of the iris and extensive PAS formation.

**Figs 72A and B:**
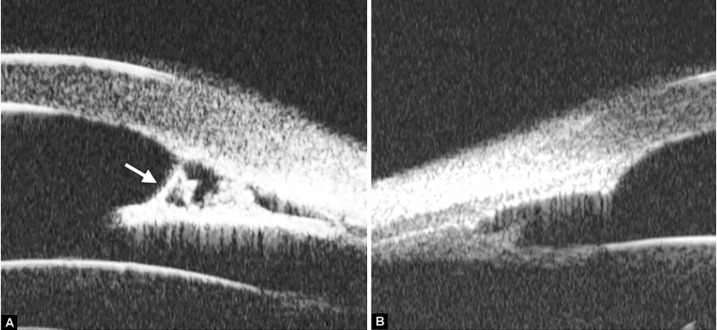
ICE syndrome. (A) There was more severe and extensive PAS in this case. In UBM images, iris stroma appears thinning apparently with fibrous tissue on the iris surface connecting to the cornea endothelium (white arrow). (B) With the further development of these fibrous tissue, atrophy of iris becomes worsen and the whole iris will adhered into cornea endothelium leaving angle closed

**Figs 73A and B:**
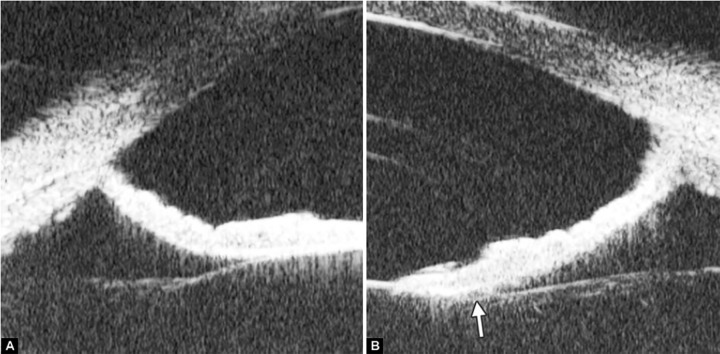
Pigment dispersion syndrome. The UBM hallmark of PDS is posterior bowing of the iris leading contact between lens, iris and zonules (white arrow in B)

Pigment Dispersion Syndrome

Pigment dispersion syndrome (PDS) is a consequence of flakes of the uveal pigment of the eye, the iris, detach from the back of the iris and float in the liquid of the eye. The flakes may obstruct the drainage outflow in the eye, thus, cause IOP rising. Mechanism of PDS is not fully understood, but in general, it is considered as a result of the proximity between the posterior iris pigment epithelium and zonular fibers and lens surface, the physical contact results in abrasion and release of the pigment granules into the posterior chamber. UBM study confirms the posterior bowing of the peripheral iris which precipitate the zonule touch, and this phenomenon is called ‘reverse pupil block’. Laser peripheral iridotomy and pilocarpine are suggested to be effective to release this block.

*Case summary:* A 36-year-old man was diagnosed as pigmentary glaucoma. Slit lamp confirmed the deepening of both anterior and peripheral chamber depth, pigmented KP and pigment granules on anterior lens surface. Gonioscopy confirmed the trabecular meshwork covered by pigmented granules.

Marfan Syndrome

Marfan syndrome is an inherited connective tissue disorder. It affects three major organ systems of the body, the auditory and circulatory system, the bones and muscles and the eyes. The eye disorders include lens dislocation, high myopia, secondary cataract, retinal detachment. Physical abnormalities include long and disproportionate extremities, long fingers, joint laxity, pectus excavatum and so on.

**Figs 74A to C:**
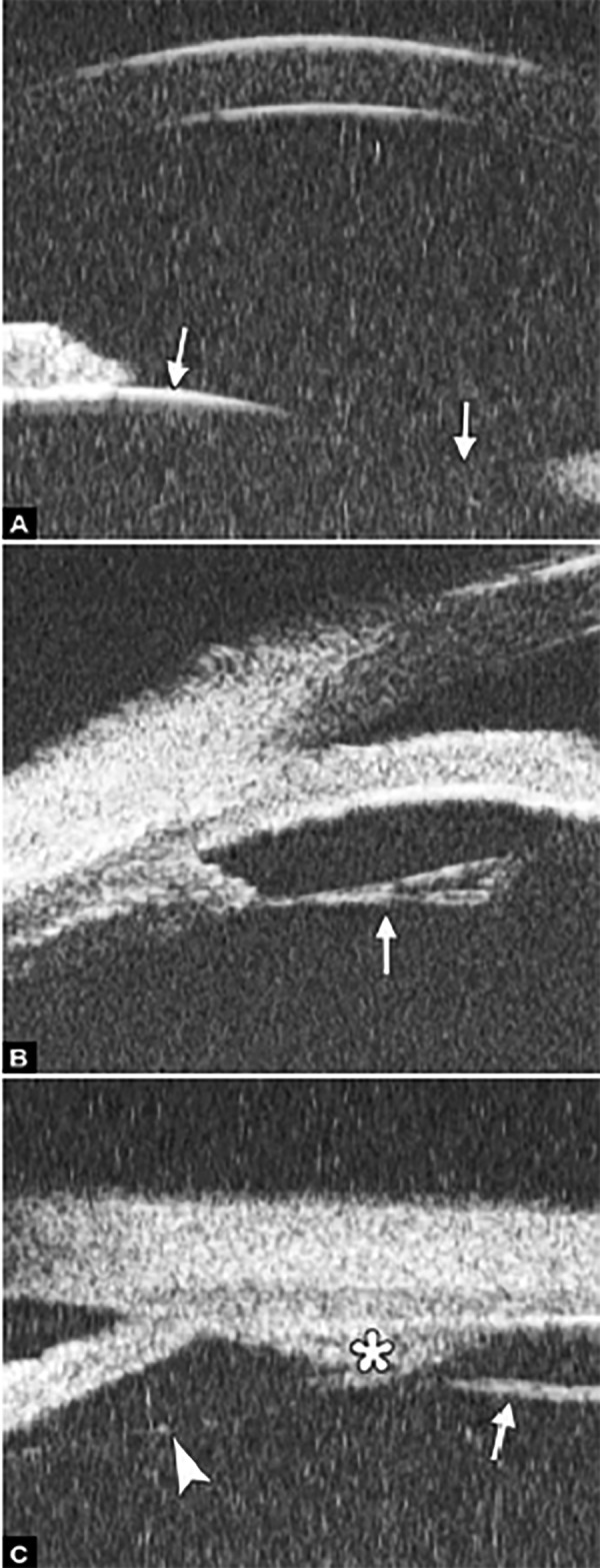
Eye disorders related to Marfan’s syndrome. (A) Shows lens subluxation with one side in normal position while the other not visible (white arrows), (B) shows the abnormally stretched zonules (white arrow), (C) shows detached peripheral retina (white arrow) and broken zonules (white arrowhead). The ciliary body appears thinning without traction of zonules (asterisk)

*Case summary 1:* A 12-year-old female was diagnosed as Marfan syndrome. Visual acuity was 0.05, slit lamp confirmed the lens displaced to nasal-inferior quadrant. UBM confirmed the changes of the lens and pars plana retinal detachment.

*Case summary* 2: A 34-year-old man was diagnosed as Marfan syndrome. Visual acuity was 0.01 in both eyes and correctable to 0.1 using -15 D eyeglasses. UBM confirmed the shape abnormality of the lens and elongation of zonules.

**Figs 75A to D:**
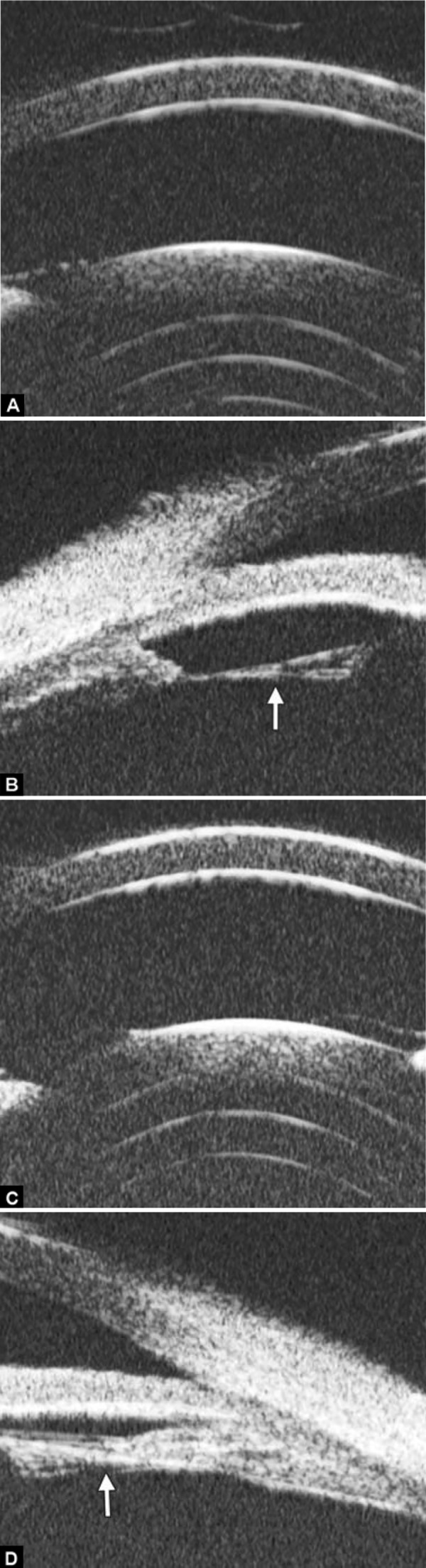
Lens and zonules changes in both eyes of a Marfan’s syndrome patient. (A and C) Abnormal curvature of anterior lens surface is identified, (B and D) show zonules are stretched for the lens dislocation (white arrow)

**Figs 76A and B:**
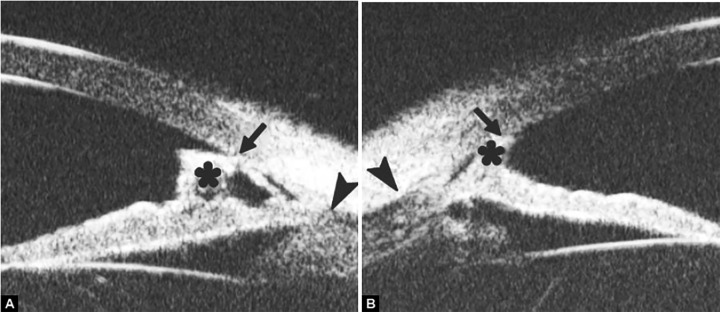
(A and B) Shows anteriorly displaced and prominent Schwalbe’s line (black arrow), high insertion of the iris into the posterior aspect of the trabecular meshwork, and the high reflective triangle of scleral spur behind the ciliary body (black arrowhead), the cross-sectional profile of the tissue band bridging peripheral iris and cornea (asterisk)

**Figs 77A to E:**
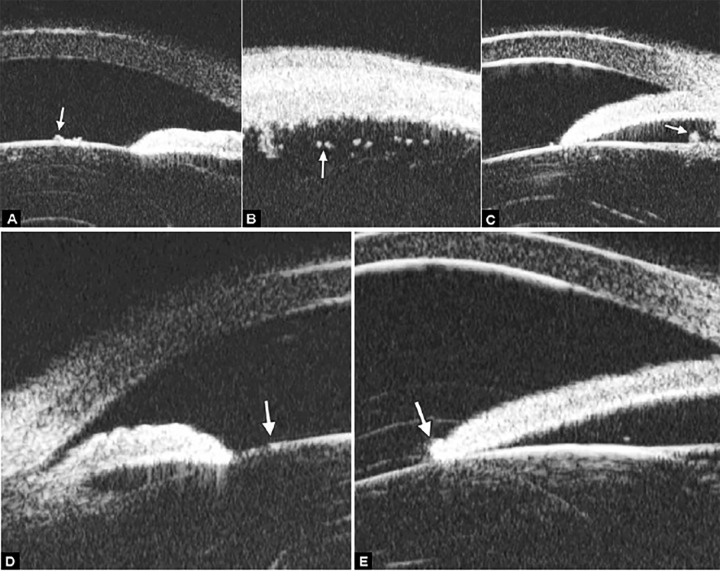
Exfoliation syndrome show the exfoliation materials lying at anterior capsule of lens, pars plana, zonules, peripheral part of lens capsule and pupil collar (white arrow)

### Axenfeld-Rieger Syndrome (ARS)

Axenfeld-Rieger syndrome (ARS) is a rare autosomal dominant disease. The ocular changes are mainly attributable to the dysgenesis of iris, trabeculae and peripheral cornea, a prominent, abnormally anteriorly displaced Schwalbe’s line, tissue strands bridging between peripheral iris and cornea and iris stromal defects. For the occlusion caused by the tissue bands across the angle and abnormal iris insertion, it is difficult to view the meshwork and scleral spur. Therefore, cross-sectional images of the anterior ocular segment may be necessary and more effective in diagnosis and evaluation of ARS.

*Case summary:* A 31-year-old man, diagnosed as ARS. Visual acuity was 0.9 in the left eye and no light perception in the right eye. The intraocular pressure (IOP) was 47 mm Hg in the right eye and 17 mm Hg in the left (measured by noncontact tomography). The cup/disk ratio of the optic nerve head reached 0.9 in both eyes. The slit lamp ocular examination showed stromal iridic defects and a prominent Schwalbe’s anterior border ring on peripheral cornea in both eyes. At around 7 to 8 O’clock of the left anterior chamber, a band of transparent tissue originating from the iris stroma was observed attached to the peripheral cornea. As a radial sectional imaging method, UBM can identify the typical pathological changes of ARS.

Exfoliation Syndrome

Exfoliation (pseudoexfoliation) syndrome is characterized by the production and progressive accumulation of a fiber extracellular material in ocular tissues. It leads to progressive glaucoma when the exfoliation materials occlude the normal outflow through trabecular meshwork.

*Case summary:* A54-year-old man presented withvisual loss for 1 month. Slit lamp examination revealed precipitations on the anterior lens surface. After dilation, the mid-peripheral region of the lens anterior surface is covered by a very thin exfoliation material. Visual acuity was 0.6, IOP 20 mm Hg. The material also presented as UBM imaging―increasing reflectivity dots presenting on the surface of ciliary body, pars plana and zonules.

